# Multi-Omics Integration Analysis of TK1 in Glioma: A Potential Biomarker for Predictive, Preventive, and Personalized Medical Approaches

**DOI:** 10.3390/brainsci13020230

**Published:** 2023-01-30

**Authors:** Chuan Shao, Pan Wang, Bin Liao, Sheng Gong, Nan Wu

**Affiliations:** 1 Graduate Institute, Chongqing Medical University, No. 1 Yixueyuan Road, Yuzhong District, Chongqing 400016, China; 2 Chongqing Institute of Green and Intelligent Technology, Chinese Academy of Sciences, Chongqing 400714, China; 3 Chongqing School, University of Chinese Academy of School, Chongqing 101408, China; 4 Department of Neurosurgery, Chongqing General Hospital, Chongqing 401147, China

**Keywords:** glioma, TK1, bioinformatics, medical informatics, multi-omics integration analysis

## Abstract

Multi-omics expression datasets obtained from multiple public databases were used to elucidate the biological function of TK1 and its effects on clinical outcomes. The Kaplan–Meier curve, a predictive nomogram mode, and the time-dependent receiver operating characteristic (ROC) curve were established to assess the role of TK1 expression in glioma prognosis. TK1 was overexpressed in glioma compared with normal samples, and patients with elevated expression of TK1 had poor overall survival. The ROC curves indicated a high diagnostic value of TK1 expression in patients of glioma; the areas under the ROC curve (AUC) were 0.682, 0.735, and 0.758 for 1 year, 3 years, and 5 years of glioma survival, respectively. For a model based on TK1 expression and other clinical characteristics, the values of AUC were 0.864, 0.896, and 0.898 for 1 year, 3 years, and 5 years, respectively. Additionally, the calibration curve indicated that the predicted and observed areas at 1 year, 3 years, and 5 years of survival were in excellent agreement. Three types of TK1 alterations—missense mutations, splice mutations, and amplifications—were identified in 25 of 2706 glioma samples. The TK1-altered group had better overall survival than the unaltered group. Single-cell function analysis showed that TK1 was positively associated with proliferation, the cell cycle, DNA repair, DNA damage, and epithelial–mesenchymal transition in glioma. Immunoinfiltration analysis indicated that TK1 expression might play different roles in low-grade glioma and glioblastoma multiforme tumor microenvironments, but TK1 expression was positively associated with activated CD4 and Th2, regardless of tumor grade. In summary, our findings identified TK1 as a novel marker for predicting clinical outcomes and a potential target for glioma.

## 1. Introduction

Glioma, originating from glial or precursor cells, is the most common and deadly brain malignancy; gliomas include astrocytic tumors (including glioblastoma), ependymoma, oligodendroglioma, oligoastrocytoma, and several rare histologies [1]. Comprehensive treatment strategies, including neurosurgical resection, radiotherapy, chemotherapy, immunotherapy, and targeted therapy, have led to only limited improvements in glioma prognosis [2]. For example, glioblastomas, accounting for 58.4% of gliomas, have a median survival time of 8 months [1]. Hence, the identification of molecular biomarkers for tumor diagnosis and therapeutic targets is both urgently needed and essential.

Thymidine kinase 1 (TK1), a special cytosolic kinase, is involved in pyrimidine metabolism, and it fluctuates during the cell cycle [3,4,5,6,7,8]. Thus, TK1 may serve as a marker of cell proliferation and cycle activity. In the 1960s, TK1 activity was found to be elevated in tumors [9]. Since then, emerging studies have shown that high levels of TK1 expression are a predictive factor in the assessment of early screening, diagnosis, progression, and treatment effects of several cancers, including lung, ovarian, colon, cervix, breast, kidney, prostate, and hematological malignancies [8,9,10,11,12,13]. Therefore, we speculated that TK1 may also be involved in malignant biological behavior in glioma. However, the precise role of TK1 and its molecular mechanisms underlying glioma progression have not been fully explored. In this study, we conducted a series of bioinformatics analyses using the public multi-omics datasets to assess the relationships of the expression level of TK1 with glioma prognosis and clinicopathological variables. In addition, we assessed the biological role of TK1 in glioma development to illustrate its therapeutic value in glioma treatment.

## 2. Materials and Methods

### 2.1. Gene Expression Analysis

The Gene Expression Profiling Interactive Analysis 2 (GEPIA2, http://gepia2.cancer-pku.cn/#index, accessed on 5 November 2021) database was used to explore differences in the mRNA expressions of TK1 genes between glioma and normal brain tissue [14]. The GTEx normal profiles and the Cancer Genome Atlas (TCGA, https://portal.gdc.cancer.gov/, accessed on 5 November 2021) tumor profiles were merged in the GEPIA2 database. We also downloaded RNA-seq and microarray expression data from the GlioVis database (http://gliovis.bioinfo.cnio.es/, accessed on 5 November 2021) [15], a powerful and large-scale tool merging data from TCGA, the Chinese Glioma Genome Atlas (CGGA; http://www.cgga.org.cn/index.jsp; accessed on 5 November 2021), and Gene Expression Omnibus (GEO, https://www.ncbi.nlm.nih.gov/geo/; accessed on 5 November 2021). We excluded pediatric tumor samples and samples from patients who received radiotherapy, chemotherapy, or immunotherapy. We also explored TK1 expression in other cancers compared with normal samples in the Oncomine (www.oncomine.org, accessed on 5 November 2021) database [16].

### 2.2. Tumor Characteristics of TK1 Expression Heterogeneity

Using the data from the GlioVis database, we assessed the relationships ofTK1 expression with WHO tumor grade, tumor type (primary or recurrent), molecular subtype, pathological subtype, and tumor regions to determine the tumor characteristics of TK1 expression heterogeneity. Primary or recurrent tumors were determined on the basis of patient medical history. Tumor regions were defined according to magnetic resonance imaging [17]. The contrast-enhanced (CE) region included a portion within the tumor’s gadolinium-enhancing core, whereas the non-contrast-enhanced (NE) involved areas of nonenhancing, FLAIR hyperintense tissue around the margins of the tumor [17].

### 2.3. Prognostic Value of TK1 Expression in Glioma

As the GlioVis database provided detailed survival data on glioma, we assessed the relationship between glioma prognosis and TK1 expression by using the Kaplan–Meier method with the logrank test. To investigate TK1 as an independent risk factor for glioma, we further assessed the relationship of TK1 expression to age, sex, WHO tumor grade, tumor type (primary or recurrent), survival time, therapy strategy (chemotherapy and/or radiotherapy), isocitrate dehydrogenase (IDH) mutation status, and 1p19q codeletion status by using the GlioVis_CGGA data. Additionally, both the time-dependent receiver operating characteristic (ROC) curve and a predictive nomogram model were established.

### 2.4. Immune Associated Analysis

Tumor Immune Estimation Resource (TIMER, https://cistrome.shinyapps.io/timer/, accessed on 20 November 2021), a comprehensive website providing the molecular characteristics of tumor immune interactions [18], was used to assess the association between TK1 expression and the abundance of immune cell infiltration levels, including for B cells, CD4+T cells, CD8+T cells, macrophages, neutrophils, and dendritic cells. Additionally, TISIDB (http://cis.hku.hk/TISIDB/, accessed on 29 November 2021) was used to evaluate the correlations between TK1 expression and immunoinhibitors, immunostimulators, and the abundance of tumor-infiltrating lymphocytes, chemokines, and receptors [19].

### 2.5. Multi-Omics Analysis

The cBio Cancer Genomics Portal (c-BioPortal, http://cbioportal.org, accessed on 5 November 2021) provides multidimensional cancer genomics data storage for exploring genetic alterations and their correlation with genes and clinical outcomes across samples [20]. In this study, we assessed the link between TK1 mutations and survival.

DNA methylation data were downloaded from the UCSC Xena platform (https://xenabrowser.net/, accessed on 5 November 2021) [21]. We first assessed the association between mRNA expression and DNA methylation levels (β-value), and then addressed the role of TK1 DNA methylation in glioma prognosis. Subsequently, the prognostic values of TK1 CpG sites were investigated. Of note, we used the Methylation450k dataset from TCGA lower-grade glioma and glioblastoma (GBMLGG), and the RNA_seq expression data are shown as normalized_log2[norm_count + 1].

### 2.6. Gene Co-Expression and Gene Set Enrichment Analysis

To investigate the functional mechanism of TK1 in gliomas, we selected the TCGA_GBMLGG dataset (*n* = 669) and obtained all genes coexpressed with TK1 from LinkedOmics (http://www.linkedomics.org/, accessed on 5 November 2021), a publicly available portal including multi-omics data from all 32 TCGA cancer types and 10 Clinical Proteomic Tumor Analysis Consortium (CPTAC) cancer cohorts [22]. Three analytical modules, including LinkFinder, LinkCompare, and LinkInterpreter, were involved in this website. LinkInterpreter assesses functional enrichment defined by the Gene Ontology, KEGG pathways, panther, reactome, and Wikipathways databases, miRNA-target, protein-protein interaction, transcription factor-target, and kinase-target networks by accessing the functional database in WebGestalt [23,24]. GO enrichment analysis and KEGG pathways analyses were performed through Gene Set Enrichment Analysis (GSEA) with the following parameters: false discovery rate (FDR) of <0.05, the minimum number of genes (size) of three, and the simulation of 500. A *p*-value < 0.05 was deemed to indicate statistical significance.

### 2.7. TK1 Associated miRNAs

We assessed the correlation between TK1 expression and miRNA expression using the microRNA_198 and mRNA-array_301 datasets from the CGGA [25]. In this study, the correlation coefficient was set at <−0.4. Next, the miRWalk database (http://mirwalk.umm.uni-heidelberg.de/, accessed on 5 June 2022) that incorporates TargetScan, miRDB, and miRTarBase data was used to predict TK1-targeting miRNAs [26]. To further verify the targeted relationship, we searched several datasets from the GEO database to confirm the differential miRNA expression between glioma and normal tissue. Finally, we also assessed the prognosis of miRNA expression and examined miRNA expression according to pathological type in the CGGA_ microRNA_198 datasets.

### 2.8. Single-Cell Function Analysis

CancerSEA (http://biocc.hrbmu.edu.cn/CancerSEA/, accessed on 10 November 2021) is a user-friendly web interface for comprehensively analyzing the gene and/or lncRNA functional states at a single-cancer-cell level [27]. These functional states include invasion, stemness, angiogenesis, metastasis, proliferation, apoptosis, epithelial-mesenchymal transition (EMT), the cell cycle, differentiation, DNA repair, DNA damage, inflammation, hypoxia, and quiescence. Herein, we reported their correlations (Spearman’s |correlation r| ≥ 0.2 and *p* < 0.05).

### 2.9. Cell Culture

Normal human astrocytes (SVG p12) and human glioma cell lines (U87 and LN229) were purchased from the American Type Culture Collection. Cell lines were cultured in DMEM with 10% fetal bovine serum and 1% penicillin and streptomycin and maintained at 37 °C in an incubator set to 5% CO_2_.

### 2.10. Protein Expression

To confirm the protein expression of TK1 in glioma, we first reported the protein ex-pression profiles from CPTAC [28,29]. Of note, TK1 protein profiles were provided for only glioblastoma, the most common type of glioma [1]. Moreover, Western blot analyses were conducted to confirm the TK1 expression on normal human astrocytes and human glioma cell lines as previously described [30]. The following antibodies were used to examine protein expression: β-actin mouse antibody (1:1000, cat. no. AF0003; Beyotime) and TK1 rabbit antibody (1:1000, cat. no. ab76495; Abcam).

### 2.11. Statistical Analysis

Categorical variables were analyzed with χ2 tests and are shown as absolute counts and proportions. The Wilcoxon test and/or the Kruskal–Wallis test were used to assess non-normally distributed continuous data. A t-test was adopted to compare two continuous groups of normally distributed data, and analysis of variance was used to compare multiple groups of normal distribution data. Spearman’s rank correlation analysis was used to examine the correlation of TK1 mRNA expression with the TK1 DNA methylation value and with TK1-associated miRNA expression. The Kaplan–Meier curve was used to assess the difference in overall survival (OS) between the group with high vs. low K1 expression. The OS was calculated as the days between diagnosis and death or the end of follow-up, whichever came first. The median values for TK1 mRNA expression, TK1 DNA methylation, and TK1-associated miRNA expression were used as the cutoffs for the high and low groups. To confirm that TK1 was an independent risk factor for glioma, we used both univariate and multivariate Cox proportional hazards models in which age, sex (male vs. female), WHO tumor grade (II vs. III vs. IV), tumor type (primary vs. recurrent), radiotherapy (no. vs. yes), chemotherapy (no. vs. yes), IDH mutation status (wildtype vs. mutant), and 1p19q codeletion status (noncodel vs. codel) were considered. Additionally, both the time-dependent ROC and a predictive nomogram model were established. To evaluate the predictive accuracy by comparing the actual and predicted outcomes, we generated correction curves. Statistical analyses were conducted with R software (version 4.2.1; http://www.R-project.org; accessed on 10 November 2022).

## 3. Results

### 3.1. TK1 Overexpression in Glioma Tissues and Other Cancers Compared with Normal Tissues

**Figure 1:**
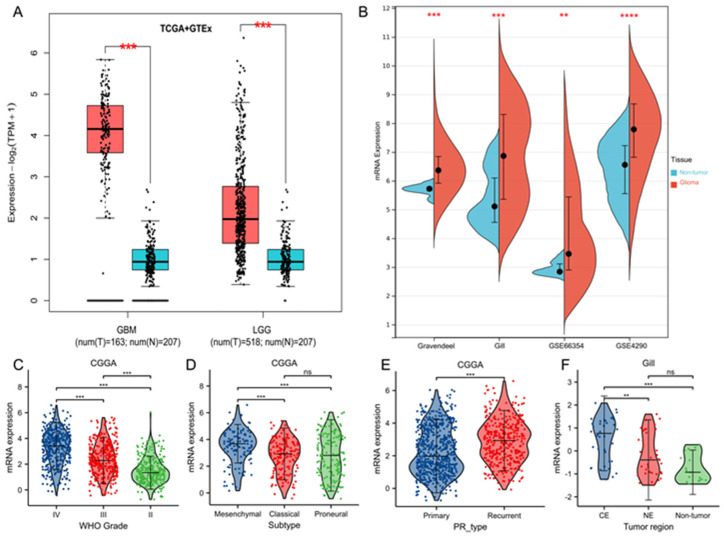
Upregulated mRNA expression of TK1 in glioma and tumor heterogeneous characteristics of TK1 expression in glioma. (**A**) TK1 is overexpressed in TCGA glioma tumors compared with the GTEx normal profiles in GEPIA2 database. (**B**) TK1 is overexpressed in glioma tumors compared with normal profiles in GEO and GlioVis databases. (**C**) TK1 expression in glioma of WHO grades II, III, and IV. (**D**) TK1 expression in subtypes of PN, ME, and CL. (**E**) TK1 expression in recurrent and primary tumors. (**F**) Different expressions of TK1 in CE and NE regions, which were defined with magnetic resonance imaging. ** *p* < 0.01, *** *p* < 0.001 and **** *p* < 0.0001. Abbreviations: PN, proneural; ME, mesenchymal; CL, classic; CE, contrast-enhanced; NE, non-contrast-enhanced; PR, primary-recurrent; ns, not significant.

To begin, the transcriptional levels of TK1 expression between glioma and normal tissues were assessed via GEPIA2 databases. TK1 was overexpressed in TCGA glioma samples compared with GTEx normal profiles (Figure 1A). To further validate the finding, we analyzed four additional datasets: GSE66354 [31], GSE4290 [32], Gravendeel microarray [33], and Gill RNA-seq file [17]; we observed consistent results (Figure 1B). The basic characteristics of the included datasets are shown in Table S1. All tumor samples were obtained before patients received any treatment. Moreover, the normal or nontumor samples were obtained from autopsies or from patients with normal pressure hydrocephalus or seizure but without oncological histories.

In addition, we measured the expression patterns of TK1 from a pan-cancer perspective through the Oncomine and GEPIA2 databases. As shown in Figure S1, TK1 expression was significantly upregulated in most tumors compared with normal tissues.

### 3.2. Tumor Characteristics of TK1 Heterogeneity in Gliomas

Human gliomas contain four molecular subtypes: neural (NE), proneural (PN), mesenchymal (ME), and classic (CL) [34,35]. Of these, the NE and PN subtypes present less aggressive behavior than the CL and ME subtypes [34,35]. TK1 expression was significantly higher in the ME subtypes than in PN subtype (Figure 1D and Figure S2D–E). No significant differences between the ME and CL subtypes were identified (Figure S2D–E). Next, we assessed the mRNA expression levels of TK1 in different grades of gliomas in four datasets and found that TK1 expression increased with the increasing glioma grade (Figure 1C and Figure S2A–C). Concerning the primary-recurrent type of tumors, we found that TK1 expression is higher in recurrent tumors than in primary tumors (Figure 1E). Additionally, TK1 expression was higher in the CE regions than in NE regions (Figure 1F), indicating intertumor heterogeneous characteristics of TK1 expression were present in GBM. Finally, we evaluated TK1 expression according to the pathological glioma subtype. As shown in Figure S3, TK1 expression varies among pathological types of gliomas.

### 3.3. Prognostic Value of TK1 Expression in Glioma

**Figure 2:**
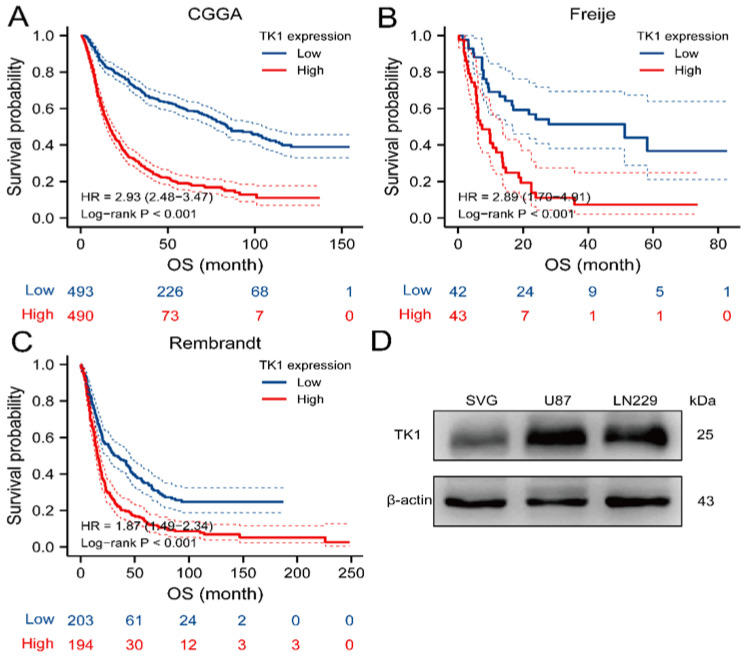
Kaplan–Meier analysis of overall survival of TK1 and TK1 protein expression in glioma cell lines and normal brain cell line. (**A**–**C**) Higher expression of TK1 is associated with poor survival in three datasets, including CGGA (**A**), Freije (**B**), and Rembrandt (**C**). (**D**) Protein expression of TK1 in glioma cell lines and normal brain cell lines. Abbreviation: OS, overall survival.

In survival analysis, the median TK1 mRNA expression value was adopted as the cutoff for the high and low groups. We identified eight datasets and then used Kaplan–Meier survival curves to assess the prognostic role of TK1 in glioma (Figure 2A–C and Figure S4A–E). A higher expression of TK1 was markedly associated with poor OS (Figure 2A–C and Figure S4A–E). Moreover, higher expression of TK1 was associated with poor prognosis in several cancers, including adrenocortical carcinoma, endocervical adenocarcinoma, kidney cancer, cervical squamous cell carcinoma, lymphoid neoplasm diffuse large B-cell lymphoma, acute myeloid leukemia, hepatocellular liver carcinoma, lung adenocarcinoma, mesothelioma, prostate adenocarcinoma, and stomach adenocarcinoma (Figure S5).

**Figure 3:**
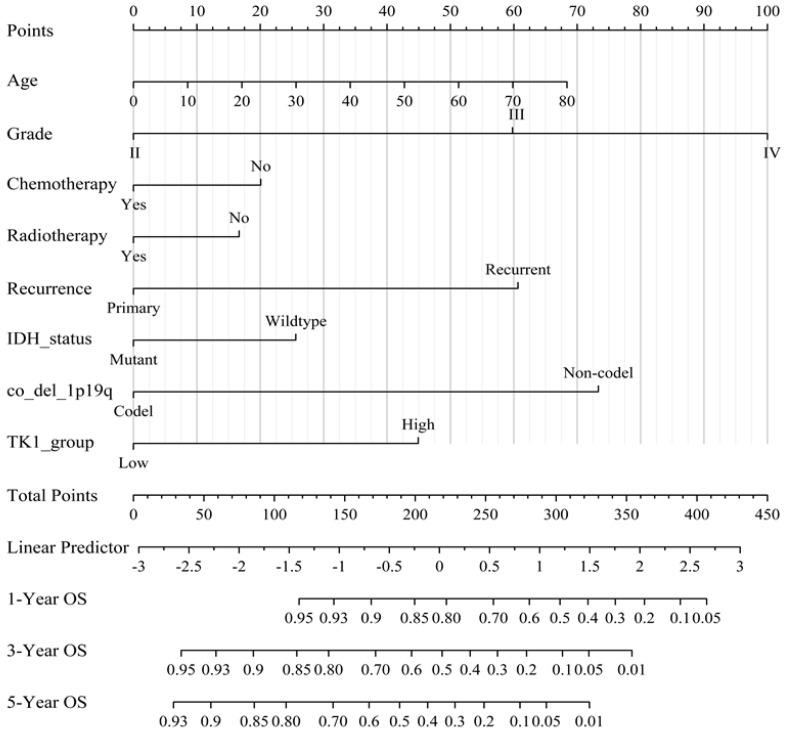
A nomogram integrating TK1 expression and clinicopathologic features predicts the clinical outcome of glioma. Abbreviations: OS, overall survival; IDH, isocitrate dehydrogenase.

**Figure 4:**
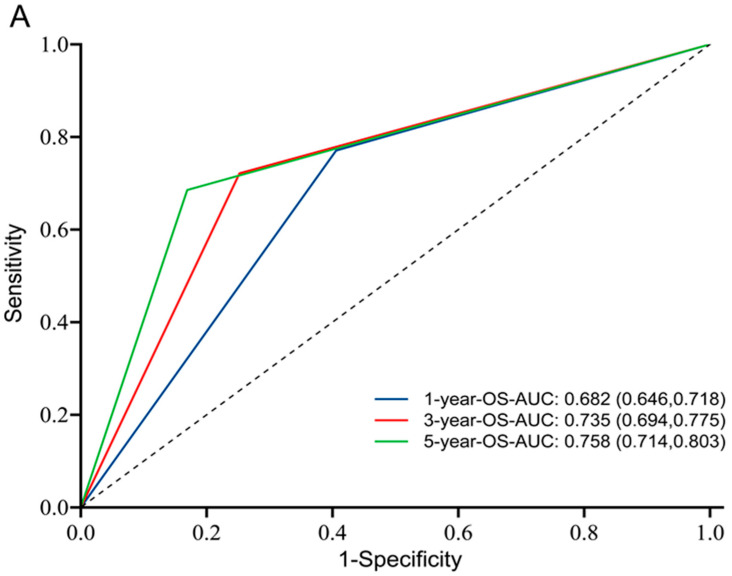

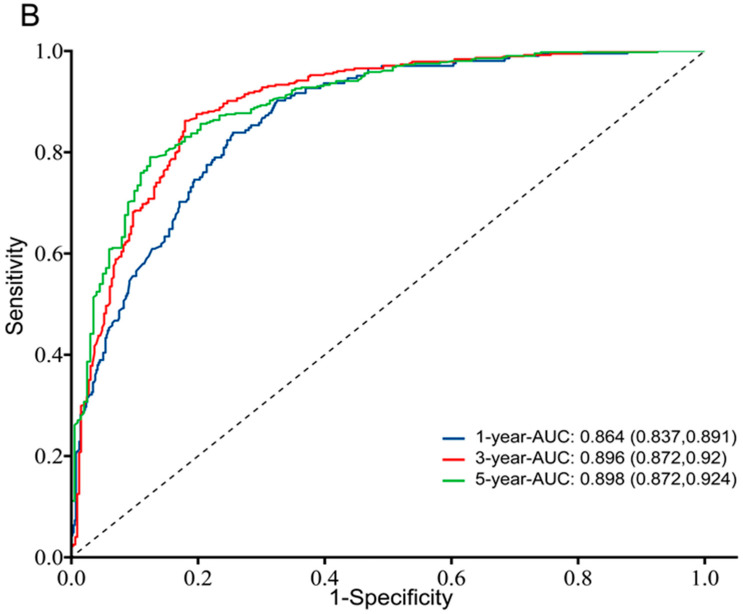
Time-dependent ROC curves in CGGA dataset. (**A**) ROC curves to predict 1-year, 3-year, and 5-year OS of glioma patients based on TK1 expression. (**B**) ROC curves to predict 1-year, 3-year, and 5-year OS of glioma patients based on TK1 expression and seven clinical features, including age, tumor type, WHO tumor grade, IDH mutation status, 1p19q codeletion status, chemotherapy, and radiotherapy. Abbreviations: OS, overall survival; IDH, isocitrate dehydrogenase; ROC, receiver operating characteristic.

Using the data from CGGA, we performed further univariate and multivariate Cox regression analyses, which showed that age, tumor type, WHO tumor grade, IDH mutation status, 1p19q codeletion status, chemotherapy, and TK1 expression were significantly associated with glioma prognosis, except for a broadline significant relationship between radiotherapy and glioma (Tables S2 and S3). We then developed a nomogram to predict 1-year, 3-year, and 5-year OS by using eight prognostic factors: age, tumor type, WHO tumor grade, IDH mutation status, 1p19q codeletion status, chemotherapy, radiotherapy, and TK1 expression (Figure 3). Time-dependent ROC curves indicated that TK1 expression had a high diagnostic value of TK1 expression in glioma patients, with an area under the curve (AUC) of 0.682, 0.735, 0.758 for 1-year, 3-year, and 5-year survival, respectively (Figure 4A). A model constructed on the basis of combination of TK1 expression and other clinical characteristics showed an AUC of 0.864, 0.896, and 0.898 for 1-year, 3-year, and 5-year OS, respectively (Figure 4B). On the basis of calibration plot, the predicted and observed 1-year, 3-year, and 5-year survival values were in excellent agreement (Figure S6). Together, these findings indicated that TK1 expression with other clinical factors performs well in predicting the prognosis of glioma.

### 3.4. Ascertainment of Protein Expression in Glioma Tissue, Glioma Cell Lines, and Other Cancers Compared with Normal Tissues

In UALCAN, the protein expression of TK1 was evaluated in 10 normal tissues and 99 glioblastoma proteomic profiles from CPTAC. TK1 protein levels were also elevated in glioblastoma tissue compared with normal tissue (Figure S7A). Moreover, we also found that TK1 was overexpressed in glioma cells compared with the SVG cell line (Figure 2D). Additionally, we found that TK1 was overexpressed in colon cancer, clear cell renal cell carcinoma, breast cancer, uterine corpus endometrial carcinoma, lung adenocarcinoma, pancreatic adenocarcinoma, head and neck squamous carcinoma, and hepatocellular carcinoma compared with normal tissue (Figure S7B–I).

### 3.5. Immune Associated Analysis

**Figure 5:**
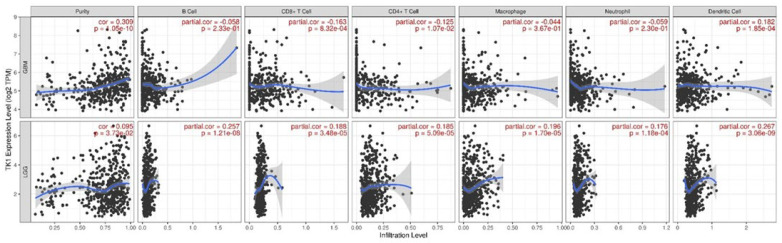
TK1 expression associated with six immune cell infiltration levels in TIMER.

Using TIMER, we explored the associations between TK1 expression and six tumor-immune cell infiltration levels. As shown in Figure 5, we observed a positive association between TK1 expression and B cell, CD4+T cell, CD8+T cell, macrophage, neutrophil, and dendritic cell infiltration levels in LGG, whereas an inverse association was identified for GBM except for dendritic cells (Figure 5).

In TISIDB, the associations between 28 tumor-infiltrating lymphocytes and TK1 expression were investigated in detail. TK1 expression was inversely associated with infiltrating levels of most kinds of lymphocytes but positively associated with activated CD4 (Act CD4) and Type 2 T (Th2) in GBM (Table S4). In LGG, TK1 expression was positively associated with infiltrating levels of most kinds of lymphocytes, particularly Act CD4, gamma delta T (Tgd), and Th2 (Table S4).

We also assessed the relationship between TK1 expression and three kinds of immunomodulators: immunoinhibitors (Table S5), immunostimulators (Table S6), and MHC molecules (Table S7). Regarding the immunostimulators, no significant association was observed between TK1 and most immunostimulators, except for C10orf54, CD276, IL6R, KLRK1, TNFRSF13C, TNFRSF4, TNFSF13, and TNFSF14 (Table S6). However, a positive association between TK1 and most immunostimulators was identified for LGG (Table S6), although some results were not significant. Regarding the immunoinhibitors, no significant association or a weak association was observed for most immunoinhibitors in GBM (Table S5). In LGG, a positive association was identified between TK1 and most immunoinhibitors, except CD160, PD-L1, CSF1R, and TIGIT (Table S5). Regarding the MHC molecules, a weak and inverse association was only observed for HLE-E in GBM (Table S7). In LGG, weak and positive associations were found between TK1 expression and all MHC molecules (Table S7).

We then examined the relationship between TK1 expression and 40 kinds of chemokines (or receptors) (Tables S8 and S9). As shown in Table S8, no significant association or a weak association was observed in GBM, except for CXCL2, CXCL5, CXCL9, CXCL14, and CXCL16. For receptors, significant inverse associations between TK1 and CCR1, CXCR3, and CX3CR1 were identified for GBM, whereas positive associations of TK1 with CCR2, CCR5, CCR7, CCR10, CXCR4, and CXCR6 were found in LGG (Table S9).

### 3.6. Multi-Omics Analysis

**Figure 6:**
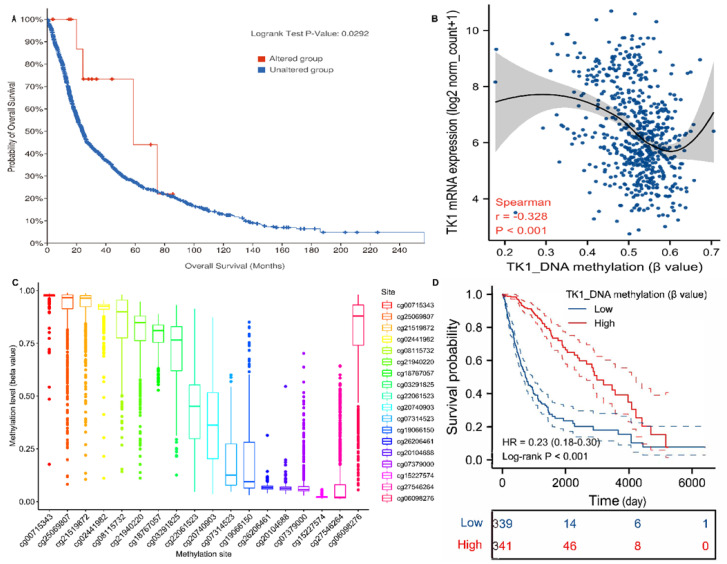
TK1 genetic alternations and DNA methylation. (**A**) TK1 alternations and the prognoses of TK1 alternations in glioma. (**B**) An inverse association between TK1 mRNA expression and TK1 DNA methylation in glioma. (**C**) Kaplan–Meier curves of TK1 DNA methylation. (**D**) The distribution of 18 TK1 DNA promoter CpG sites in glioma.

Genetic alterations of TK1 in glioma were explored with cBioPortal. Overall, three kinds of alterations, including missense mutations, splice mutations, and amplifications, were identified in 25 of 2706 glioma samples (Figure S8). Additionally, glioma patients with genetic alterations had better OS (Figure 6A).

By assembling the UCSC Xena databases, we found a negative correlation (Spearman’s coefficients = −0.328, *p* < 0.001) between TK1 expression and TK1 DNA methylation (Figure 6B). Further analysis showed that lower TK1 methylation correlated with favorable OS (Figure 6C). The distribution of 18 TK1 CpG sites is shown in Figure 6D, and a significant and inverse correlation was observed between methylation at most CpG sites and TK1 mRNA expression, except for the cg02441982, cg08115732, and cg15227574 sites (Figure S9). Kaplan–Meier plots also indicated that higher methylation levels of 15 CpG sites were associated with better OS, except for the cg26206461, cg20104688, and cg26206461 sites (Figure S10).

### 3.7. Co-Expressed Gene and Gene Set Enrichment Analysis

**Figure 7:**
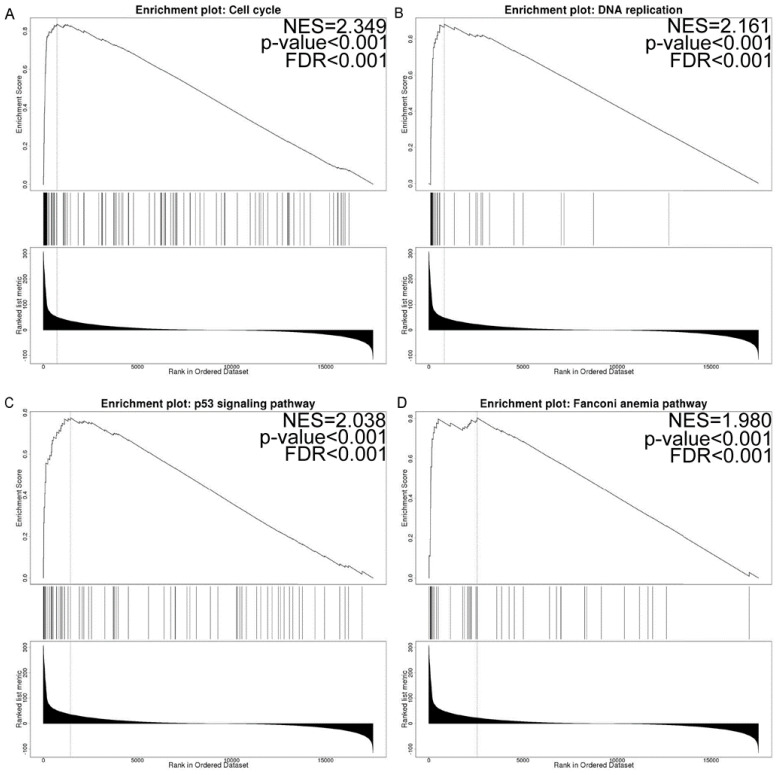
GSEA results. (**A**) Cell cycle. (**B**) DNA replication. (**C**) p53 signaling pathway. (**D**) Fanconi anemia pathway. Abbreviations: GSEA, gene set enrichment analysis; NES, normalized enrichment score; FDR, false discovery rate.

By assembling the LinkedOmics database, we selected the TCGA_GBMLGG sets to identify the TK1-associated coexpressed genes and predicted their function. The top 50 TK1-associated coexpressed negative and positive genes are exhibited in Figure S11. The GO functional annotations suggested that these genes were involved predominantly in biological regulation, responses to stimulus, metabolic processes, cell communication, cell proliferation, reproduction, growth, and other functions at the biological process level (Figure S11). At the cell component level, these genes were mainly enriched in the membrane, membrane-enclosed lumen, cytosol, protein-containing complex, endomembrane system, vesicle, cell projection, cytoskeleton, endosome, mitochondrion, chromosome, extracellular matrix, ribosome, microbody, lipid droplet, and other functions (Figure S11). At the molecular function, protein binding, ion binding, nucleic acid binding, hydrolase activity, nucleotide binding, transferase activity, transporter activity, enzyme regulator activity, lipid binding, carbohydrate-binding, molecular adaptor activity, electron transfer activity, translation regulator activity, oxygen binding, and other functions were enriched (Figure S11). Additionally, 50 key pathways associated with TK1 were identified via GSEA analysis (Table S10). The top four enrichment pathways were DNA replication, the cell cycle, the p53 signaling pathway, and the Fanconi anemia pathway (Figure 7 and Table S10).

### 3.8. Candidate miRNA Prediction

**Figure 8:**
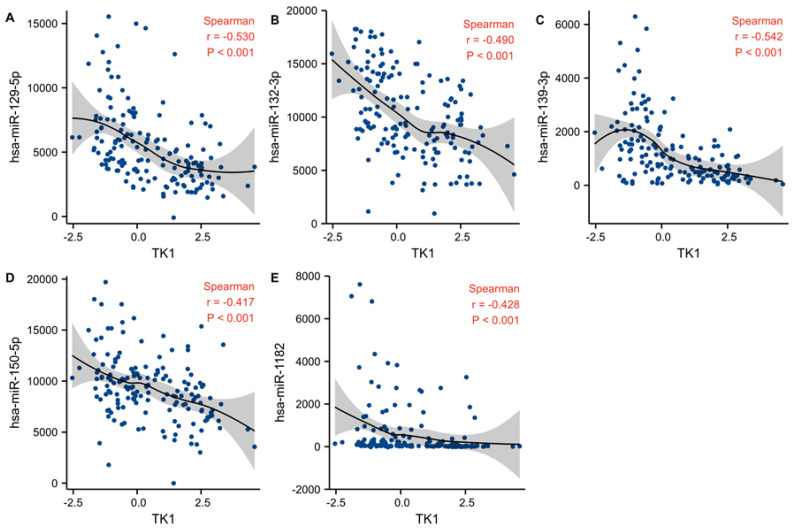
An inverse association between TK1 and hsa-miR-129-5p (**A**), hsa-miR-132-3p (**B**), hsa-miR-139-3p (**C**), hsa-miR-150-5p (**D**), and hsa-miR-1182 (**E**).

**Figure 9:**
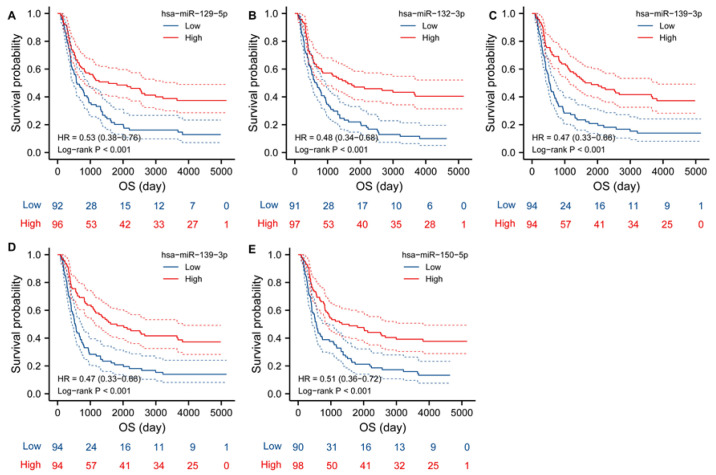
Kaplan–Meier analysis of overall survival of five TK1-associated miRNAs, including hsa-miR-129-5p (**A**), hsa-miR-132-3p (**B**), hsa-miR-139-3p (**C**), hsa-miR-150-5p (**D**), and hsa-miR-1182 (**E**). Abbreviation: OS, overall survival.

Next, to verify whether miRNAs might regulate TK1 expression, we identified miRNAs negatively correlated with TK1 in the microRNA_198 and mRNA-array_301 datasets from the CGGA. Using the datasets from CGGA, we identified 12 miRNAs negatively correlated with TK1 expression (Spearman’s coefficients ≤ −0.40, *p* < 0.05, Table S11 and Figure 8). We next examined gene–miRNA interactions with a focus on predicting miRNAs in the miRWalk database. Seven miRNAs were further identified (Table S11). Then, we assessed the differential expressions of these miRNAs between tumors and normal samples in the GSE90603, GSE103228, GSE165937, GSE25631, GSE138764, GSE158284, GSE13030, and GSE135819 datasets. We found that hsa-miR-1182, hsa-miR-129-5p, hsa-miR-132-3p, hsa-miR-139-3p, and hsa-miR-150-5p each had lower expression in glioma than in normal samples (Table S12). Additionally, we evaluated the prognostic values of miRNA expression in CGGA_ microRNA_array_198. The median values of TK1-associated miRNA expression were defined as the cutoffs for high and low categories. We found that lower expressions of hsa-miR-1182, hsa-miR-129-5p, hsa-miR-132-3p, hsa-miR-139-3p, and hsa-miR-150-5p were associated with shorter OS in glioma (Figure 9). Collectively, our findings indicated that these miRNAs might regulate TK1 expression in glioma. Additionally, we assessed the expressions of five miRNAs according to pathological glioma type. As shown in Figure S12, their expressions varied among types.

### 3.9. Single-Cell Function Analysis

**Figure 10:**
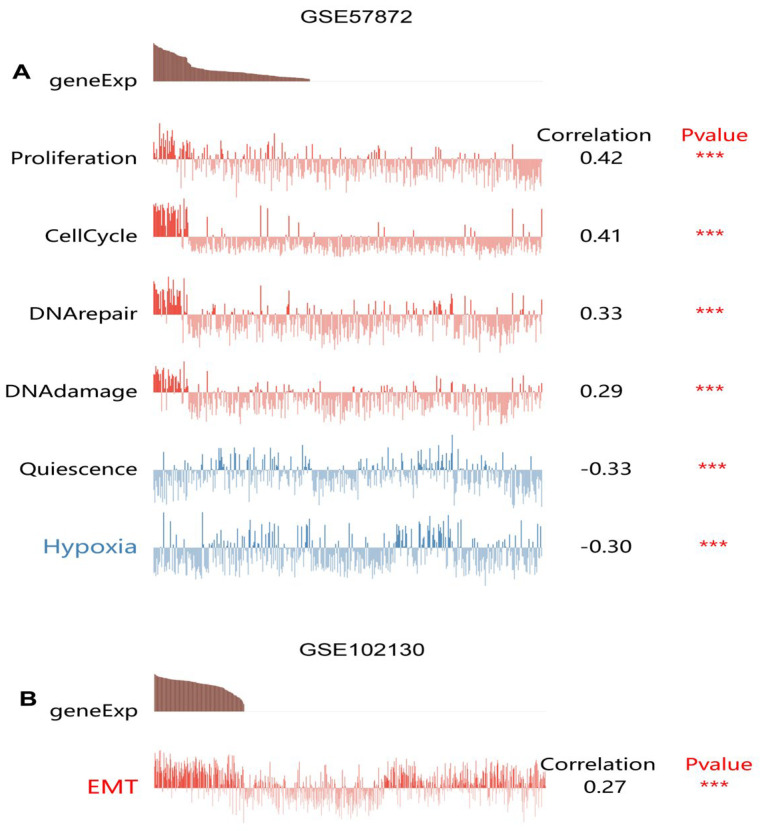
Single-cell function analysis of TK1 expression in GSE57872 (**A**) and GSE 102130 (**B**) datasets. *** *p* < 0.001. Abbreviation: EMT, epithelial–mesenchymal transition.

Using CancerSEA, we identified two GEO datasets (GSE57872 and GSE 102130) for assessing the functional characteristics of TK1 at a single-cancer-cell level in glioma. Figure 10 shows that TK1 was positively associated with proliferation (Spearman’s coefficients = 0.42, *p* < 0.001), the cell cycle (Spearman’s coefficients = 0.41, *p* < 0.001), DNA repair (Spearman’s coefficients = 0.33, *p* < 0.001), DNA damage (Spearman’s coefficients = 0.29, *p* < 0.001), and EMT (Spearman’s coefficients = 0.27, *p* < 0.001), but negatively correlated with quiescence (Spearman’s coefficients = −0.33, *p* < 0.001) and hypoxia (Spearman’s coefficients = −0.30, *p* < 0.001).

## 4. Discussion

Recent studies have suggested an association between TK1 and the development of several tumors, including lung cancer, thyroid carcinoma, prostate cancer, and pancreatic cancer [5,6,7,8]. However, the role of TK1 expression and its biological function in glioma had not been elucidated. Here, we first comprehensively explored TK1 profiles of expression, prognosis, functional signaling, and immune infiltration in glioma.

In our analysis of transcriptional data collected from TCGA, GTEx, and GEO, we discovered that TK1 expression was higher in glioma tissue samples and cell lines than in normal samples or normal cell lines. Further survival analysis indicated that patients with higher levels of TK1 expression had shorter OS. To confirm the TK1 protein expression, we assessed the expression of TK1 in normal cells and tumor cell lines. The results showed that TK1 was overexpressed in U87 and LN229 cell lines compared with SVG. Additionally, we found that missense mutations, splice mutations, and amplifications were involved in glioma samples, and our survival analysis showed that the TK1-altered group had better OS than the group with these alterations. Moreover, high TK1 expression was observed in many other tumors and was found to be associated with poor prognosis, as shown in Figure S2. Collectively, these findings suggested that TK1 might serve as a prognostic biomarker for glioma.

DNA methylation is a common method of regulating gene transcription. To our knowledge, TK1 methylation has not been addressed in previous studies. For the first time, we revealed that a negative association between TK1 methylation and TK1 mRNA expression in glioma and methylation levels could determine OS in patients with glioma. Further analysis identified a similar relationship between TK1 mRNA expression and TK1 CpG sites. Notably, we did not identify the methylation levels of TK1 between tumor and normal samples; thus, further exploration is still required.

MiRNAs are small ncRNA molecules of 19–25 nucleotides that can target the 30 untranslated regions of their target mRNA to inhibit degradation and translation [36]. Liu et al. have found that miR-34a-5p suppresses the expression of TK1 in thyroid carcinoma cell lines by binding to its 3′ untranslated regions [4]. However, on the basis of the bioinformatic analysis from the TCGA_GBMLGG dataset, we did not confirm such a relationship between TK1 and miR-34a-5p (Spearman’s coefficients = 0.033, *p* = 0.45). According to our predefined criteria, we identified that hsa-miR-1182, hsa-miR-129-5p, hsa-miR-132-3p, hsa-miR-139-3p, and hsa-miR-150-5p miRNAs may regulate TK1 expression in glioma by using the microRNA_198 and mRNA-array_301 datasets from the CGGA. Among the five miRNAs, hsa-miR-132-3p, hsa-miR-139-3p, and hsa-miR-150-5p were downregulated in glioma, and their overexpression has been found to prevent cell proliferation, colony formation, and tumor growth in gliomas [37,38,39]. Additionally, miR-150-5p regulates stem cell characteristics, thereby inhibiting the progression of glioma through effects on the Wnt/β-catenin pathway [37]. Regarding the other two miRNAs, hsa_miR-129-5p activates the AKT signal transduction pathway in renal cell carcinoma, which plays a role in cell proliferation, metabolism, angiogenesis, and metastasis [40,41]. Moreover, hsa-miR-1182 overexpression inhibits cell proliferation, colony formation, and invasion of bladder cancer [42], gastric cancer [43], and non-small cell lung cancer [44]. Collectively, these miRNAs may regulate TK1 expression in glioma.

Recent findings suggest that TK1 expression influences the proliferation of lung cancer, thyroid carcinoma, prostate cancer, melanoma, and pancreatic cancer cells [4,5,6,7,8], but inconsistent results on migration and invasion have been reported in lung cancer, prostate cancer, and thyroid carcinoma [4,6,7]. In our study, both GSEA enrichment analyses and single-cell function analysis indicated that the functions of TK1 and associated genes were involved in proliferation, the cell cycle, DNA repair, DNA damage, EMT, and the p53 signaling pathway. Moreover, ASF1B, which ranked first among the top 50 TK1-associated coexpressed positive genes (Spearman’s coefficients = 0.947, *p* < 0.001) in glioma, has been shown to be an oncogene functioning partially through the P53-mediated EMT signaling pathway in lung cancer cells [45]. Additionally, knockdown of the expression of BIRC5 (Spearman’s coefficients = 0.939, *p* < 0.001) and RRM2 (Spearman’s coefficients = 0.936, *p* < 0.001), two additional top 50 TK1-associated coexpressed positive genes, inhibits the migration, proliferation, and apoptosis of glioma cells [46,47]. Together, these findings suggest that TK1 might act as an oncogene in glioma.

Growing evidence indicates that immunological cells (both antitumorigenic and protumorigenic) in the tumor microenvironment play central roles in tumorigenesis [48]. A previous study by Cai et al. has found that TK1 expression is significantly associated with infiltrating levels of “CD4 memory resting T cells”, “CD4 memory activated T cells”, “follicular helper T cells”, Tregs, “naïve B cells”, and “activated dendritic cells” in hepatocellular carcinoma [8]. In this study, we analyzed TK1 expression with infiltration levels of six tumor-immune cells by using the TIMER database. Interestingly, inverse associations between TK1 expression and infiltrating levels of B cells, CD4+T cells, CD8+T cells, neutrophils, macrophages, and dendritic cells were observed in LGG, whereas a positive association was found for GBM. Using the TISIDB database, we also investigated the association between 28 tumor-infiltrating lymphocytes and TK1 expression in detail. Similarly, two opposite trends were identified in most types of lymphocytes between LGG and GBM, except activated CD4 and Th2 cells. Concerning the immunomodulators (i.e., immunoinhibitors, immunostimulators, and MHC molecules) and chemokines (or receptors), we found that TK1 was inversely associated with most molecules in GBM but not in LGG. Collectively, these findings indicated that TK1 might play different roles in the LGG and GBM tumor microenvironments.

Evidence has shown that serum TK1 is a reliable biomarker for detecting malignant tumors in cancer screening [49,50]. In a routine health examination of 56,286 people aged 13–86 years, Sven Skog et al. found that TK1 was more sensitive than CEA and AFP in detecting malignant tumors, and about 30% more sensitivity was achieved with a combination of TK1, CEA, and AFP [49]. Another survey with 35,365 participants also showed that the concentration of TK1 protein in the serum is a reliable indicator of early cancer progression risk [50]. Currently, early diagnosis of glioma is extremely difficult. Therefore, future studies are warranted to clarify the diagnostic role of TK1 expression in cerebrospinal fluid samples for the early detection of gliomas.

Several study limitations should be acknowledged. First, we did not fully clarify the role of TK1 expression in the different pathological subtypes of gliomas because few datasets provided data on pathological subtypes, and various pathological subtypes were defined in the included datasets. As suggested in Figure 1 and Figure S2, TK1 expression exhibited some inter- and intratumor heterogeneity in gliomas. Therefore, further detailed analysis of the specific pathological subtypes may provide new insights into glioma. Second, we were unable to address the roles of chemotherapy types, radiotherapy doses, obesity, smoking status, and other parameters in the association between TK1 and glioma prognosis because no detailed data were provided by CGGA. Finally, our study provides only preliminary protein expression validation in glioma tissue and glioma cells compared with normal samples. Thus, the specific mechanisms and gene–gene and miRNA–gene interactions remain to be confirmed through additional in vitro and in vivo experiments.

## 5. Conclusions

In conclusion, the current study comprehensively explored the malignant properties of TK1 in glioma through multiple levels of bioinformatics analysis. The findings provide critical insights for further investigation of TK1 as a potential target.

## Data Availability

The datasets presented in this study can be found in online repositories/Supplementary Material (GEPIA2, http://gepia2.cancer-pku.cn/#index; TCGA, https://portal.gdc.cancer.gov/; CGGA (http://www.cgga.org.cn/; GlioVis database, http://gliovis.bioinfo.cnio.es/; GEO, https://www.ncbi.nlm.nih.gov/geo/; CCLE, https://portals.broadinstitute.org/ccle/; Oncomine, www.oncomine.org; TIMER, https://cistrome.shinyapps.io/timer/; c-BioPortal, http://cbioportal.org; UCSC Xena platform, https://xenabrowser.net/; LinkedOmics, http://www.linkedomics.org/; UALCAN, http://ualcan.path.uab.edu/index.html; miRWalk data-base, http://mirwalk.umm.uni-heidelberg.de/; CancerSEA, http://biocc.hrbmu.edu.cn/CancerSEA/; and TISIDB, http://cis.hku.hk/TISIDB/). Additionally, the datasets generated during and/or analyzed during the current study are available from the corresponding authors on reasonable request.

## Supplementary Materials

The following supporting information can be downloaded at: https://www.mdpi.com/article/10.3390/brainsci13020230/s1, Figure S1: Transcription levels of TK1 in other tumors comparing normal samples in Oncomine (A) and GEPIA2 databases (B). The text in red represents higher expression of TK1 in tumors than in normal samples in Figure S1B. The text in green represents lower expression of TK1 in tumors than in normal samples in Figure S1B. The text in black represents no significant difference between tumors and normal samples in Figure S1B. Abbreviations: ACC, adrenocortical carcinoma; BLCA, bladder urothelial carcinoma; BRCA, breast invasive carcinoma; CESC, cervical squamous cell carcinoma and endocervical adenocarcinoma; CHOL, cholangio carcinoma; COAD, colon adenocarcinoma; DLBC, lymphoid neoplasm diffuse large B-cell lymphoma; ESCA, esophageal carcinoma; HNSC, head and neck squamous cell carcinoma; KICH, kidney chromophobe; KIRC, kidney renal clear cell carcinoma; KIRP, kidney renal papillary cell carcinoma; LAML, acute myeloid leukemia; LIHC, liver hepatocellular carcinoma; LUAD, lung adenocarcinoma; LUSC, lung squamous cell carcinoma; MESO, mesothelioma; OV, ovarian serous cystadenocarcinoma; PAAD, pancreatic adenocarcinoma; PCPG, pheochromocytoma and paraganglioma; PRAD, prostate adenocarcinoma; READ, rectum adenocarcinoma; SARC, sarcoma; SKCM, skin cutaneous melanoma; STAD, stomach adenocarcinoma; TGCT, testicular germ cell tumors; THCA, thyroid carcinoma; THYM, thymoma; UCEC, uterine corpus endometrial carcinoma; UCS, uterine carcinosarcoma; UVM, uveal melanoma; Figure S2: Tumor-heterogeneous characteristics of TK1 expression in glioma. (A–C) TK1 expression in glioma of WHO grade II, III, and IV. (D–F) TK1 expression in subtype of PN, ME, and CL. * *p* < 0.05, ** *p* < 0.01, and *** *p* < 0.001. Abbreviations: PN, proneural; ME, mesenchymal; CL, classic; ns, not significant; Figure S3: TK1 expression in various pathological subtypes. A. CGGA. B. Rembrandt. C. Gravendeel.; Figure S4: Kaplan–Meier analysis of overall survival of TK1 in five datasets. A. TCGA. B. Vital. C. Phillips. D. Gravendeel. E. Lee Y. Abbreviation: OS, overall survival; Figure S5: Kaplan–Meier analysis of overall survival of 12 cancers, including ACC (A), CESC (B), DLBC (C), KICH (D), KIRC (E), KIRP (F), LAML (G), LIHC (H), LUAD (I), MESO (J), PRAD (K), and STAD (L). Abbreviations: ACC, adrenocortical carcinoma; CESC, cervical squamous cell carcinoma and endocervical adenocarcinoma; DLBC, lymphoid neoplasm diffuse large B-cell lymphoma, KICH, kidney chromophobe; KIRC, kidney renal clear cell carcinoma; KIRP, kidney renal papillary cell carcinoma; LAML, acute myeloid leukemia; LIHC, liver hepatocellular carcinoma; LUAD, lung adenocarcinoma; MESO, mesothelioma; PRAD, prostate adenocarcinoma; STAD, stomach adenocarcinoma; Figure S6: Calibration curve for nomogram to predict 1-year (A), 3-year (B), and 5-year (C) OS. Figure S7: Upregulated protein expression of TK1 in glioblastoma (A), breast cancer (B), clear cell renal cell carcinoma (C), colon cancer (D), head and neck squamous carcinoma (E), hepatocellular carcinoma (F), lung adenocarcinoma (G), pancreatic adenocarcinoma (H), and UCEC (I) compared with normal tissue. * *p* < 0.05, ** *p* < 0.01, and *** *p* < 0.001. Abbreviations: RCC, renal cell carcinoma; UCEC, uterine corpus endometrial carcinoma; Figure S8: TK1 genetic alternations; Figure S9: The relationship between TK1 mRNA expression and 18 TK1 DNA-promoter CpG sites in glioma. A. cg00715343. B. cg02441982. C. cg03291825. D. cg06098276. E. cg07314523. F. cg07379000. G. cg08112732. H. cg15227574. I. cg18757057. J. cg1906650. K. cg20104688. L. cg20740903. M. cg21519872. N. cg21940220. O. cg22061523. P. cg25069807. Q. cg26206461. R. cg27546264; Figure S10: Kaplan-Meier curves of 18 TK1 DNA promoter CpG sites in glioma. A. cg00715343. B. cg02441982. C. cg03291825. D. cg06098276. E. cg07314523. F. cg07379000. G. cg08112732. H. cg15227574. I. cg18757057; J. cg1906650. K. cg20104688. L. cg20740903. M. cg21519872. N. cg21940220. O. cg22061523. P. cg25069807. Q. cg26206461. R. cg27546264; Figure S11: The coexpression genes of TK1 and GO functional annotation based on the TCGA_GBMLGG set. A. The heat map of top 50 negative associations with TK1. B. The heat map of top 50 positive associations with TK1. C-E. GO functional annotation involved biological process (C), cellular component (D), and molecular function (E); Figure S12: Five miRNA expression according to the pathological types of glioma. A. hsa-miR-129-5p. B. hsa-miR-132-3p. C. hsa-miR-139-3p. D. hsa-miR-150-5p. E. hsa-miR-1182. Abbreviations: GBM, glioblastoma; rGBM, recurrent glioblastoma; AA, anaplastic astrocytoma; rAA, recurrent anaplastic astrocytoma; AOA, anaplastic oligoastrocytoma; A, astrocytoma; rA, recurrent astrocytoma; O, oligodendroglioma; OA, oligoastrocytoma; AO, anaplastic oligodendrolgioma; rAO, recurrent anaplastic oligodendroglioma. Table S1: Basic characteristics of included datasets; Table S2: Clinicopathological parameters according to TK1 expression; Table S3: Univariate and multivariate analyses of clinicopathological parameters related to survival; Table S4: Spearman correlations between tumor-infiltrating lymphocytes and TK1 expression; Table S5: Spearman correlations between immunoinhibitors and TK1 expression; Table S6: Spearman correlations between immunostimulators and TK1 expression; Table S7: Spearman correlations between MHCs and TK1 expression; Table S8: Spearman correlations between chemokines and TK1 expression; Table S9: Spearman correlations between receptors and TK1 expression; Table S10: Enrichment results for GSEA; Table S11: miRNA list; Table S12: Different expression of miRNAs between tumor and normal tissue in GEO datasets.
