# Supplementary material for: Multi-Omics Integration Analysis of TK1 in Glioma: A Potential Biomarker for Predictive, Preventive, and Personalized Medical Approaches
Source: Brain Sci. 2023 Jan 30;13(2):230. doi: 10.3390/brainsci13020230 (PMC9954725; doi:10.3390/brainsci13020230)
Supplement: Supplementary file 1 [file brainsci-13-00230-s001.zip › brainsci-2117556-supplementary.pdf]

Supplementary file

Table S1 Basic characteristics of included datasets.

Database	Datasets	Tumor (N)	Normal or non-tumors (N)
GEPIA2	TCGA	681	207
GlioVis	Gravendeel	276	8
GlioVis	Gill[a]	75	17
GEO	GSE66354[b]	7	13
GEO	GSE4290[c]	157	23

Abbreviation: N, number.

[a] Seventeen nonneoplastic/normal brain samples were collected from patients with normal pressure hydrocephalus or seizure, but without no oncological history.

[b] Thirteen normal brain samples were obtained from autopsy and patients with epilepsy, but without no oncological history. We excluded pediatric tumor samples in the GSE66354.

[c] Twenty-three nonneoplastic/normal brain samples were collected from with seizure, but without no oncological history.

Table S2. clinicopathological parameters according to TK1 expression

Characteristic*	TK1 expression		P-value
	Low group (n=509)	High group (n=509)	
Age (y), median (Q1-Q3)	41.00 (34.00-49.00)	44.00 (36.00-55.00)	<0.001
Gender, n (%)			0.848
Male	299 (58.74%)	302 (59.33%)	
Female	210 (41.26%)	207 (40.67%)	
WHO Grade, n (%)			<0.001
II	239 (46.95%)	52 (10.32%)	
III	173 (33.99%)	161 (31.94%)	
IV	97 (19.06%)	291 (57.74%)	
Recurrence, n (%)			<0.001
Primary	367 (72.10%)	284 (56.24%)	
Recurrent	142 (27.90%)	221 (43.76%)	
IDH status, n (%)			<0.001
Wildtype	147 (30.43%)	288 (59.63%)	
Mutant	336 (69.57%)	195 (40.37%)	
1p/19q codeletion, n (%)			<0.001
Non-codel	302 (68.33%)	426 (85.54%)	
Codel	140 (31.67%)	72 (14.46%)	
Radiotherapy, n (%)			0.071
No	94 (19.83%)	70 (15.32%)	
Yes	380 (80.17%)	387 (84.68%)	
Chemotherapy, n (%)			<0.001
No	166 (36.17%)	109 (24.17%)	
Yes	293 (63.83%)	342 (75.83%)	

Abbreviation: y, year; n, number; IDH, isocitrate dehydrogenase. *There were 5, 4, 52, 78, 87, 108 subjects missing the data for WHO grade, recurrence, IDH status, 1p/19q codeletion, radiotherapy, and chemotherapy, respectively.

Table S3. Univariate and multivariate analyses of clinicopathological parameters related to survival.

Characteristics	Univariate		Multivariate	
	HR (95% CI)	p-value	HR (95% CI)	p-value
Age	1.03(1.02, 1.04)	<0.0001	1.01 (1.01, 1.02)	0.0006
Gender				
Male	1.0 (reference)		1.0 (reference)	
Female	1.02 (0.86, 1.20)	0.8521	0.88 (0.73, 1.06)	0.1703
WHO Grade				
II	1.0 (reference)		1.0 (reference)	
III	2.82 (2.18, 3.65)	<0.0001	2.37 (1.74, 3.22)	<0.0001
IV	7.92 (6.17, 10.16)	<0.0001	4.09 (2.91, 5.74)	<0.0001
Recurrence				
Primary	1.0 (reference)			
Recurrent	2.24 (1.90, 2.64)	<0.0001	2.37 (1.96, 2.86)	<0.0001
IDH status				
Wildtype	1.0 (reference)		1.0 (reference)	
Mutant	0.32 (0.27, 0.38)	<0.0001	0.69 (0.56, 0.86)	0.0012
1p/19q codeletion				
Non-codel	1.0 (reference)			
Codel	0.22 (0.17, 0.29)	<0.0001	0.36 (0.26, 0.49)	<0.0001
Radiotherapy				
No	1.0 (reference)		1.0 (reference)	
Yes	1.02 (0.81, 1.28)	0.8604	0.77 (0.59, 1.00)	0.0504
Chemotherapy				
No	1.0 (reference)		1.0 (reference)	
Yes	1.52 (1.26, 1.85)	<0.0001	0.74 (0.59, 0.93)	0.0108
TK1 expression				
Low	1.0 (reference)		1.0 (reference)	
High	3.15 (2.65, 3.74)	<0.0001	1.85 (1.49, 2.30)	<0.0001

Abbreviation: HR, hazard ratio; 95% CI, 95% confidence interval; IDH, isocitrate dehydrogenase.

Table S4 Spearman correlations between tumor-infiltrating lymphocytes and TK1 expression.

Description	GBM		LGG	
	rho	P-value	rho	P-value
Activated CD8 T cell	0.128	1.01E-01	0.28	5.93E-11
Central memory CD8 T cell	0.121	1.20E-01	0.176	4.63E-05
Effector memory CD8 T cell	-0.129	9.76E-02	0.12	5.74E-03
Activated CD4 T cell	0.598	<2.2E-16	0.698	<2.2E-16
Central memory CD4 T cell	0.025	7.49E-01	0.163	1.67E-04
Effector memory CD4 T cell	-0.16	3.94E-02	-0.098	2.44E-02
T follicular helper cell	-0.018	8.20E-01	0.06	1.68E-01
Gamma delta T cell	0.152	5.12E-02	0.437	<2.2E-16
Type 1 T helper cell	-0.125	1.07E-01	0.016	7.11E-01
Type 17 T helper cell	-0.358	2.54E-06	-0.146	7.77E-04
Type 2 T helper cell	0.35	4.20E-06	0.433	<2.2E-16
Regulatory T cell	-0.04	6.09E-01	0.192	9.02E-06
Activated B cell	-0.197	1.09E-02	-0.025	5.65E-01
Immature B cell	-0.284	2.17E-04	-0.006	8.91E-01
Memory B cell	0.05	5.19E-01	0.257	2.31E-09
Natural killer cell	-0.234	2.47E-03	0.061	1.61E-01
CD56bright natural killer cell	0.025	7.44E-01	0.157	2.86E-04
CD56dim natural killer cell	-0.09	2.47E-01	0.197	5.22E-06
Myeloid derived suppressor cell	-0.076	3.28E-01	0.129	2.94E-03
Natural killer T cell	-0.196	1.15E-02	0.225	1.82E-07
Activated dendritic cell	-0.075	3.39E-01	0.147	7.15E-04
Plasmacytoid dendritic cell	-0.256	8.84E-04	-0.124	4.26E-03
Immature dendritic cell	-0.303	7.65E-05	-0.173	6.59E-05
Macrophage	-0.062	4.31E-01	0.042	3.20E-01
Eosinophil	-0.19	1.42E-02	-0.163	1.73E-04
Mast cell	-0.125	1.08E-01	0.073	9.10E-02
Monocyte	-0.019	8.13E-01	0.066	1.31E-01
Neutrophil	-0.284	2.22E-04	0.112	1.02E-02

Table S5 Spearman correlations between immunoinhibitors and TK1 expression.

Description	GBM		LGG	
	rho	P-value	rho	P-value
ADORA2A	0.029	7.15E-01	0.13	2.80E-03
CD160	-0.22	4.48E-03	-0.189	1.22E-05
CD244	-0.108	1.65E-01	0.042	3.31E-01
PD-L1	-0.187	1.62E-02	-0.097	2.59E-02
CD96	-0.038	6.31E-01	0.23	8.89E-08
CSF1R	-0.157	4.31E-02	-0.091	3.62E-02
CTLA4	-0.099	2.05E-01	NA	NA
HAVCR2	-0.123	1.15E-01	0.115	8.02E-03
IDO1	0.068	3.82E-01	0.311	3.26E-13
IL10	-0.077	3.27E-01	0.056	2.02E-01
ILR10RB	0.178	2.15E-02	0.41	<2.2E-16
KDR	0.01	9.01E-01	0.19	1.05E-05
LAG3	0.167	3.15E-02	0.316	1.25E-13
LGALS9	-0.033	6.72E-01	0.194	6.95E-06
PD-1	0.079	3.10E-01	0.214	6.74E-07
PD-L2	-0.21	6.74E-03	0.134	2.07E-03
PVRL2	0.076	3.31E-01	0.215	6.29E-07
TGFB1	0.061	4.34E-01	0.094	3.05E-02
TIGIT	-0.12	1.25E-01	-0.103	1.75E-02
VTCN1	0.275	3.53E-04	0.139	1.37E-03

Abbreviation: NA, not available.

Table S6 Spearman correlations between immunostimulator and TK1 expression

Description	GBM		LGG	
	rho	P-value	rho	P-value
C10orf54	-0.284	2.23E-04	-0.19	1.10E-05
CD27	0.012	8.80E-01	0.214	6.86E-07
CD276	0.306	6.70E-05	0.494	<2.2E-16
CD28	0.103	1.86E-01	0.187	1.46E-05
CD40	0.039	6.21E-01	0.121	5.42E-03
CD48	0.037	6.35E-01	0.297	3.63E-12
CD70	0.008	9.15E-01	NA	NA
CD80	-0.001	9.90E-01	NA	NA
CD86	-0.146	5.97E-02	0.027	5.39E-01
CXCL12	-0.022	7.75E-01	0.019	6.57E-01
CXCR4	0.048	5.39E-01	0.111	1.04E-02
ENTPD1	-0.095	2.23E-01	-0.046	2.96E-01
ICOSLG	-0.077	3.26E-01	0.158	2.66E-04
IL2RA	-0.051	5.12E-01	0.193	8.17E-06
IL6	-0.002	9.76E-01	0.13	2.69E-03
IL6R	0.352	3.91E-06	-0.133	2.15E-03
KLRK1	0.217	5.01E-03	0	9.93E-01
LTA	-0.004	9.63E-01	NA	NA
MICB	0.002	9.80E-01	0.317	1.12E-13
NT5E	-0.001	9.92E-01	0.024	5.74E-01
PVR	0.077	3.27E-01	0.172	7.01E-05
TMEM173	0.023	7.64E-01	0.233	6.58E-08
TMIGD2	-0.138	7.69E-02	0.017	6.97E-01
TNFRSF13C	0.227	3.35E-03	0.001	9.76E-01
TNFRSF14	-0.188	1.52E-02	0.17	8.56E-05
TNFRSF18	-0.006	9.40E-01	NA	NA
TNFRSF25	-0.149	5.52E-02	-0.029	5.07E-01
TNFRSF4	0.163	3.65E-02	0.255	3.16E-09
TNFRSF8	-0.069	3.77E-01	0.033	4.45E-01
TNFSF13	-0.211	6.38E-03	-0.084	5.32E-02
TNFSF13B	-0.124	1.11E-01	0.154	3.83E-04
TNFSF14	-0.267	5.16E-04	NA	NA
TNFSF4	-0.09	2.51E-01	-0.164	1.55E-04
TNFSF9	-0.064	4.10E-01	-0.097	2.50E-02
ULBP1	-0.004	9.62E-01	0.169	9.38E-05

Abbreviation: NA, not available.

Table S7 Spearman correlations between MHCs and TK1 expression.

Description	GBM		LGG	
	rho	P-value	rho	P-value
B2M	-0.092	2.40E-01	0.265	6.72E-10
HLA-A	0.065	4.02E-01	0.28	6.35E-11
HLA-B	-0.041	6.02E-01	0.265	6.51E-10
HLA-C	-0.015	8.50E-01	0.228	1.28E-07
HLA-DMA	-0.072	3.58E-01	0.21	1.15E-06
HLA-DMB	-0.082	2.92E-01	0.158	2.77E-04
HLA-DOA	-0.137	7.78E-02	0.182	2.67E-05
HLA-DOB	-0.061	4.36E-01	0.169	9.88E-05
HLA-DPA1	-0.049	5.32E-01	0.244	1.34E-08
HLA-DPB1	-0.021	7.86E-01	0.249	6.69E-09
HLA-DQA1	-0.12	1.24E-01	0.263	8.67E-10
HLA-DQA2	0.062	4.29E-01	0.172	7.11E-05
HLA-DQB1	-0.085	2.78E-01	0.246	1.13E-08
HLA-DRA	-0.049	5.29E-01	0.215	6.05E-07
HLA-DRB1	-0.054	4.90E-01	0.245	1.17E-08
HLE-E	-0.207	7.57E-03	0.088	0.0437
HLE-F	-0.09	2.50E-01	0.217	5.07E-07
HLE-G	-0.081	3.02E-01	0.181	2.71E-05
TAP1	0.048	5.42E-01	0.264	8.33E-10
TAP2	-0.104	1.82E-01	0.181	2.81E-05
TAPBP	-0.145	6.18E-02	0.146	7.41E-04

Table S8 Spearman correlations between chemokines and TK1 expression.

Expression	GBM		LGG	
	rho	P-value	rho	P-value
CCL2	-0.114	1.45E-01	-0.013	7.64E-01
CCL3	-0.054	4.90E-01	-0.16	2.22E-04
CCL4	-0.024	7.55E-01	-0.139	1.30E-03
CCL5	0.015	8.47E-01	0.235	4.64E-08
CCL7	-0.098	2.10E-01	NA	NA
CCL8	0.056	4.74E-01	-0.054	2.14E-01
CCL13	0.029	7.08E-01	NA	NA
CCL14	0.005	9.50E-01	-0.033	4.48E-01
CCL18	0.013	8.72E-01	-0.003	9.44E-01
CCL19	-0.109	1.60E-01	-0.041	3.42E-01
CCL20	-0.117	1.33E-01	NA	NA
CCL22	-0.172	2.70E-02	-0.049	2.56E-01
CCL26	0.077	3.23E-01	NA	NA
CCL28	-0.172	2.64E-02	NA	NA
CX3CL1	-0.1	2.02E-01	-0.209	1.26E-06
CXCL1	-0.144	6.49E-02	-0.076	8.03E-02
CXCL2	-0.233	2.56E-03	-0.151	4.93E-04
CXCL3	-0.17	2.85E-02	-0.235	4.97E-08
CXCL5	-2.06	7.81E-03	-0.324	2.64E-14
CXCL6	-0.055	4.79E-01	NA	NA
CXCL8	-0.104	1.83E-01	-0.067	1.23E-01
CXCL9	0.358	1.20E-17	0.066	4.01E-01
CXCL10	0.069	3.78E-01	0.291	1.14E-11
CXCL11	-0.012	8.75E-01	0.271	2.62E-10
CXCL12	-0.022	7.75E-01	0.019	6.57E-01
CXCL13	0.019	8.04E-01	0.007	8.69E-01
CXCL14	-0.269	4.73E-04	-0.077	7.52E-02
CXCL16	-0.207	7.55E-03	0.04	3.56E-01

Abbreviation: NA, not available.

Table S9 Spearman correlations between receptor and TK1 expression.

Expression	GBM		LGG	
	rho	P-value	rho	P-value
CCR1	-0.157	4.37E-02	0.067	1.24E-01
CCR2	-0.134	8.46E-02	0.147	6.78E-04
CCR5	-0.145	6.24E-02	0.194	7.03E-06
CCR6	-0.141	6.96E-02	0.005	9.01E-01
CCR7	0.076	3.31E-01	0.253	3.68E-09
CCR10	-0.035	6.53E-01	0.197	5.40E-06
CXCR1	-0.142	6.85E-02	-0.084	5.22E-02
CXCR2	-0.221	4.37E-03	0.053	2.26E-01
CXCR3	0.027	7.27E-01	NA	NA
CXCR4	0.048	5.39E-01	0.111	1.04E-02
CXCR6	0.047	5.44E-01	0.216	5.53E-07
CX3CR1	-0.32	2.92E-05	-0.136	1.69E-03

Abbreviation: NA, not available.

Table S10 Enrichment results for GSEA.

geneSet	Description	Enrichment Score	NES	p-Value	FDR	Size	Leading Edge Number
hsa04110	Cell cycle	0.83641195	2.348627671	<2.2e-16	<2.2e-16	118	41
hsa03030	DNA replication	0.883243012	2.161058801	<2.2e-16	<2.2e-16	36	23
hsa04115	p53 signaling pathway	0.774854389	2.038117984	<2.2e-16	<2.2e-16	69	25
hsa03460	Fanconi anemia pathway	0.803899669	1.979991553	<2.2e-16	<2.2e-16	44	25
hsa05322	Systemic lupus erythematosus	0.696978583	1.975052084	<2.2e-16	<2.2e-16	121	73
hsa03430	Mismatch repair	0.861396733	1.932955992	<2.2e-16	<2.2e-16	23	14
hsa00240	Pyrimidine metabolism	0.685417719	1.930600277	<2.2e-16	<2.2e-16	96	31
hsa03440	Homologous recombination	0.806284164	1.903307099	<2.2e-16	<2.2e-16	34	13
hsa04713	Circadian entrainment	-0.621858634	-2.047386631	<2.2e-16	<2.2e-16	94	39
hsa05033	Nicotine addiction	-0.735606951	-2.087136403	<2.2e-16	<2.2e-16	40	27
hsa04911	Insulin secretion	-0.622903272	-2.106668958	<2.2e-16	<2.2e-16	85	32
hsa04724	Glutamatergic synapse	-0.622126578	-2.166961804	<2.2e-16	<2.2e-16	111	48
hsa03050	Proteasome	0.735298397	1.876303224	<2.2e-16	4.90E-04	44	32
hsa05169	Epstein-Barr virus infection	0.619416256	1.842954645	<2.2e-16	6.13E-04	197	104
hsa03410	Base excision repair	0.782972311	1.859934539	<2.2e-16	6.68E-04	33	13
hsa05150	Staphylococcus aureus infection	0.724975973	1.863006561	<2.2e-16	7.35E-04	52	37
hsa04114	Oocyte meiosis	0.642291624	1.798158673	<2.2e-16	0.001243852	118	20
hsa04070	Phosphatidylinositol signaling system	-0.578960395	-1.982647691	<2.2e-16	0.001614056	97	43
hsa04728	Dopaminergic synapse	-0.554198798	-1.993664616	<2.2e-16	0.001883066	131	48
hsa04730	Long-term depression	-0.612150424	-1.957611061	<2.2e-16	0.002092295	56	25
hsa04512	ECM-receptor interaction	0.648426582	1.777052111	<2.2e-16	0.00210001	80	42
hsa04727	GABAergic synapse	-0.592068634	-1.999315487	<2.2e-16	0.002259679	88	37
hsa04020	Calcium signaling pathway	-0.52919345	-1.974535891	<2.2e-16	0.002353832	181	77
hsa00562	Inositol phosphate metabolism	-0.579607353	-1.938056599	<2.2e-16	0.002636292	74	23
hsa05032	Morphine addiction	-0.555060588	-1.889600893	<2.2e-16	0.00308138	89	35
hsa00983	Drug metabolism	0.640651375	1.750085018	0.002610966	0.003332016	74	14
hsa04925	Aldosterone synthesis and secretion	-0.547345463	-1.86638194	<2.2e-16	0.003452287	96	33
hsa05330	Allograft rejection	0.737481269	1.740478849	<2.2e-16	0.003675018	35	26
hsa04742	Taste transduction	-0.558367199	-1.833726745	<2.2e-16	0.003987668	81	24
hsa00650	Butanoate metabolism	-0.688199529	-1.834310616	<2.2e-16	0.004236898	27	12
hsa05031	Amphetamine addiction	-0.566002755	-1.842529098	<2.2e-16	0.004268282	68	26
hsa03420	Nucleotide excision repair	0.7003576	1.722787289	<2.2e-16	0.004410021	45	16
hsa00071	Fatty acid degradation	-0.619666616	-1.842966934	<2.2e-16	0.004573159	42	13
hsa04024	cAMP signaling pathway	-0.492363342	-1.852937232	<2.2e-16	0.004635238	195	67
hsa04970	Salivary secretion	-0.541935731	-1.819980035	<2.2e-16	0.004812279	86	30
hsa05332	Graft-versus-host disease	0.723234595	1.714983254	0.002898551	0.004981691	37	24
hsa05222	Small cell lung cancer	0.614789962	1.704275774	<2.2e-16	0.005953529	93	23
hsa05320	Autoimmune thyroid disease	0.675670423	1.706623721	<2.2e-16	0.006034766	50	26
hsa04914	Progesterone-mediated oocyte maturation	0.610565102	1.701762584	<2.2e-16	0.006090029	94	19
hsa04723	Retrograde endocannabinoid signaling	-0.497828762	-1.797162769	<2.2e-16	0.00614474	140	49
hsa04927	Cortisol synthesis and secretion	-0.567773569	-1.788272107	<2.2e-16	0.006456225	64	18
hsa04720	Long-term potentiation	-0.576123815	-1.788390957	<2.2e-16	0.006779036	67	33
hsa04080	Neuroactive ligand-receptor interaction	-0.466628088	-1.771769027	<2.2e-16	0.007532262	274	104
hsa00510	N-Glycan biosynthesis	0.670864907	1.680187506	<2.2e-16	0.008500476	48	24
hsa04672	Intestinal immune network for IgA production	0.677054855	1.681805739	<2.2e-16	0.008686406	45	30
hsa04218	Cellular senescence	0.582116246	1.675232251	<2.2e-16	0.009003794	155	31
hsa04261	Adrenergic signaling in cardiomyocytes	-0.486743253	-1.751349312	<2.2e-16	0.009169711	144	51
hsa01212	Fatty acid metabolism	-0.593604534	-1.74140224	<2.2e-16	0.009729172	44	14
hsa04961	Endocrine and other factor-regulated calcium reabsorption	-0.569577874	-1.711651325	0.007246377	0.012955491	47	16
hsa00480	Glutathione metabolism	0.643538128	1.651172478	<2.2e-16	0.014817672	54	17

Abbreviation: GSEA, gene set enrichment analysis; NES, normalized enrichment score; FDR, false discovery rate.

Table S11 miRNA list.

Gene	miRNA	Spearman's coefficients	p-value	Predicted by miRwalk
TK1	hsa-let-7c-5p	-0.441	<0.000001	Yes
TK1	hsa-miR-1182	-0.428	<0.000001	Yes
TK1	hsa-miR-1245	-0.442	<0.000001	No
TK1	hsa-miR-128-3p	-0.465	<0.000001	No
TK1	hsa-miR-1288-3p	-0.43	<0.000001	No
TK1	hsa-miR-129-5p	-0.53	<0.000001	Yes
TK1	hsa-miR-1301-3p	-0.511	<0.000001	Yes
TK1	hsa-miR-132-3p	-0.49	<0.000001	Yes
TK1	hsa-miR-139-3p	-0.542	<0.000001	Yes
TK1	hsa-miR-139-5p	-0.579	<0.000001	No
TK1	hsa-miR-150-5p	-0.417	<0.000001	Yes
TK1	hsa-miR-151-5p	-0.417	<0.000001	No

Table S12 Different expression of miRNAs between tumor and normal tissue in GEO datasets.

miRNA	GSE90603		GSE103228		GSE165937		GSE25631		GSE158284		GSE13030		GSE138764		GSE135189	
	logFC	P.Value	logFC	P.Value	logFC	P.Value	logFC	P.Value	logFC	P.Value	logFC	P.Value	logFC	P.Value	logFC	P.Value
hsa-miR-1182	-1.117	7.46E-06													-2.275	5.15E-05
hsa-miR-129-5p	-2.687	2.13E-04	-3.766	2.21E-04	-5.26	1.01E-07									-3.717	2.58E-09
hsa-miR-132-3p	-1.175	3.70E-03			-2.131	8.82E-06					-1.46	7.06E-06	-3.007	3.52E-11		
hsa-miR-139-3p	-2.929	6.53E-06			-4.161	4.67E-09	-3.166	1.49E-04	-5.812	5.04E-08			-2.156	5.55E-07	-4.515	5.88E-08
hsa-miR-150-5p											-1.147	2.13E-04				

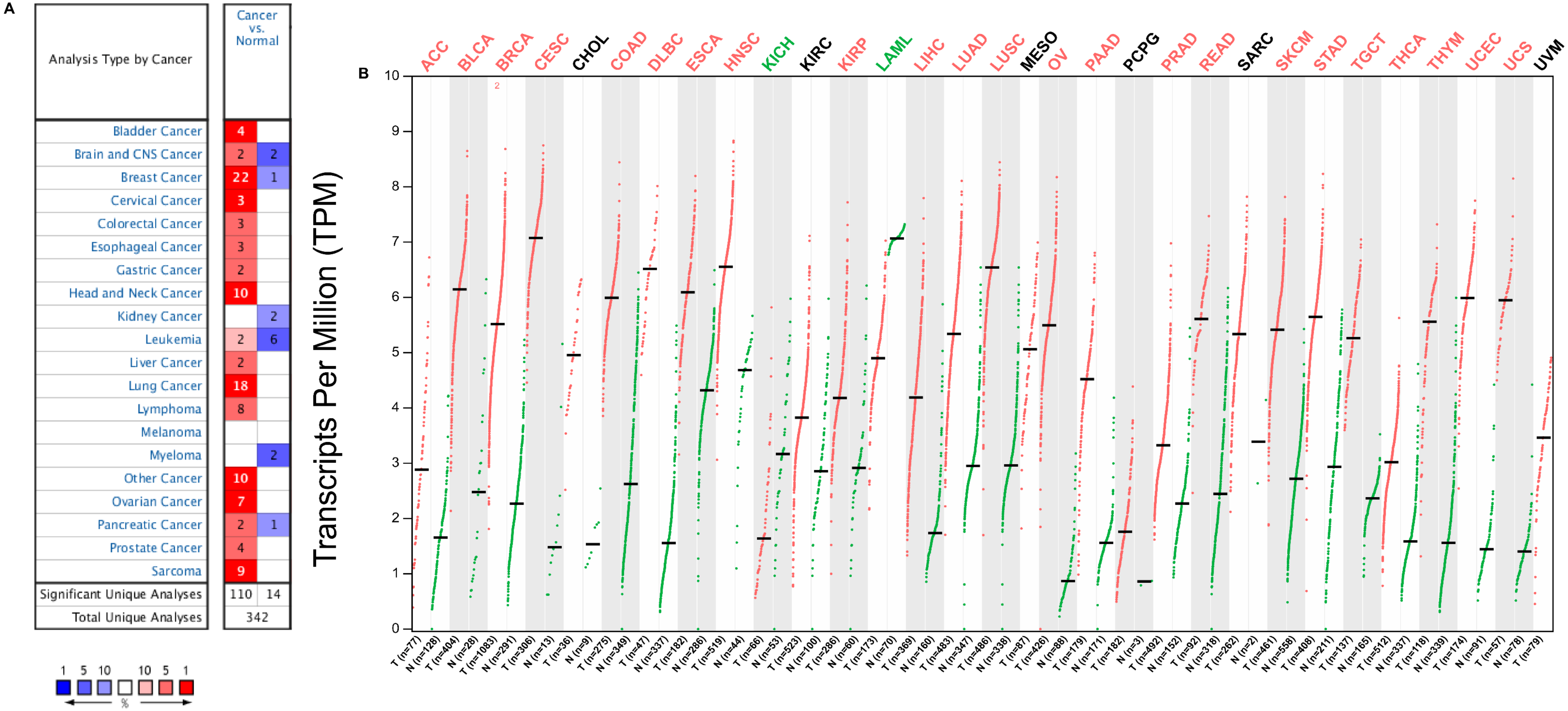

Figure S1. Transcription levels of TK1 in other tumors comparing normal samples in Oncomine (A) and GEPIA2 database (B). The texts in red represent higher expression of TK1 in tumors than in normal samples in Figure S1B. The texts in green represent lower expression of TK1 in tumors than in normal samples in Figure S1B. The texts in black represent no significant difference between tumors and normal samples in Figure S1B. Abbreviation: ACC, adrenocortical carcinoma; BLCA, bladder urothelial carcinoma; BRCA, breast invasive carcinoma; CESC, cervical squamous cell carcinoma and endocervical adenocarcinoma; CHOL, cholangio carcinoma; COAD, colon adenocarcinoma; DLBC, lymphoid neoplasm diffuse large B-cell lymphoma; ESCA, esophageal carcinoma; HNSC, head and Neck squamous cell carcinoma; KICH, kidney Chromophobe; KIRC, kidney renal clear cell carcinoma; KIRP, kidney renal papillary cell carcinoma; LAML, acute myeloid leukemia; LIHC, liver hepatocellular carcinoma; LUAD, lung adenocarcinoma; LUSC, lung squamous cell carcinoma; MESO, mesothelioma; OV, ovarian serous cystadenocarcinoma; PAAD, pancreatic adenocarcinoma; PCPG, pheochromocytoma and paraganglioma; PRAD, prostate adenocarcinoma; READ, rectum adenocarcinoma; SARC, sarcoma; SKCM, skin cutaneous melanoma; STAD, stomach adenocarcinoma; TGCT, testicular germ cell tumors; THCA, thyroid carcinoma; THYM, thymoma; UCEC, uterine corpus endometrial carcinoma; UCS, uterine carcinosarcoma; UVM, uveal melanoma.

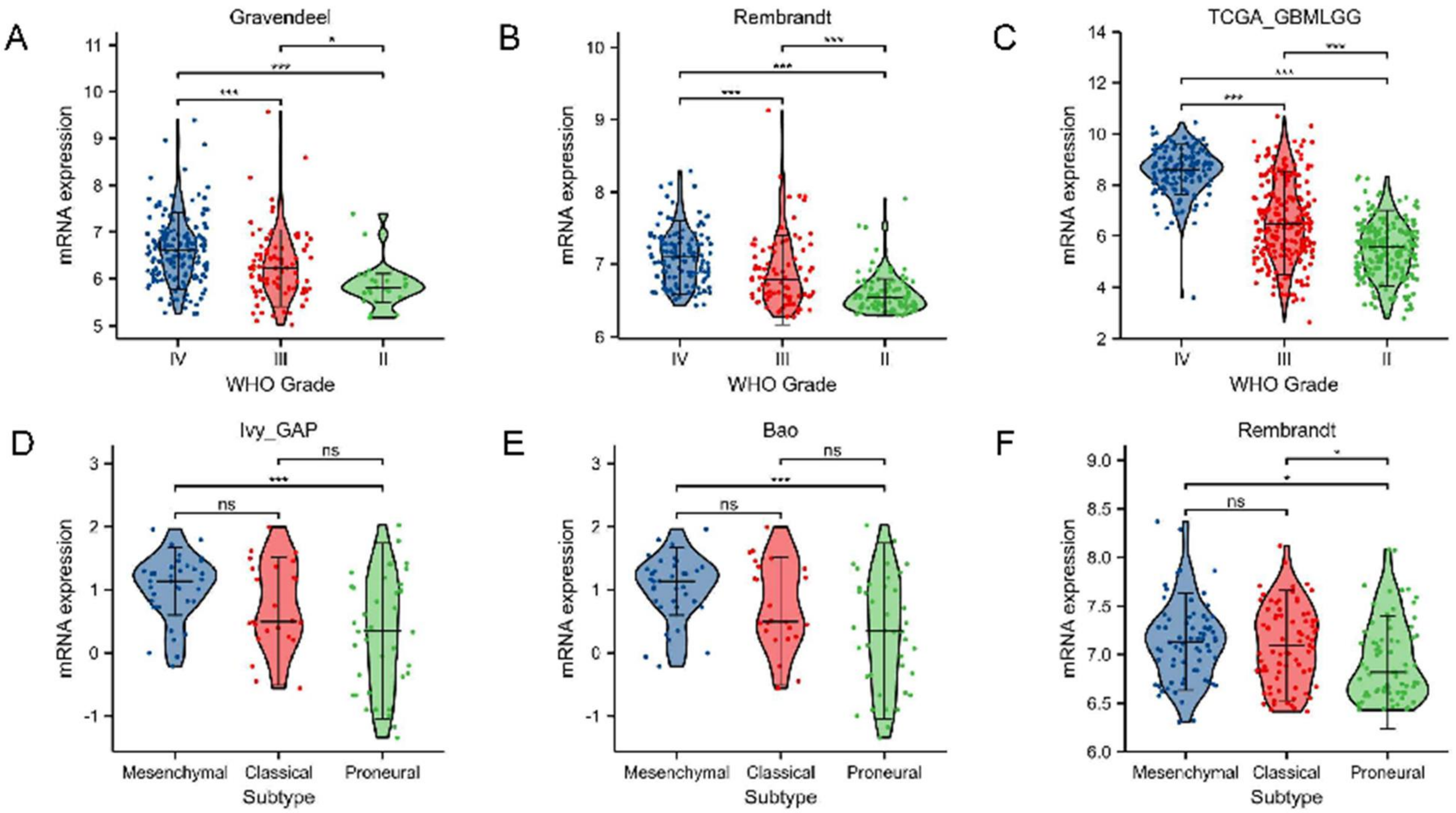

Figure S2. Tumor heterogeneous characteristics of TK1 expression in glioma. A-C. TK1 expression in glioma of WHO grade II, III, and IV. D-F. TK1 expression in subtype of PN, ME, and CL. *p < 0:05, **p < 0:01, and ***p < 0:001. Abbreviation: PN, pro-neural; ME, mesenchymal; CL, classic; ns, not significant.

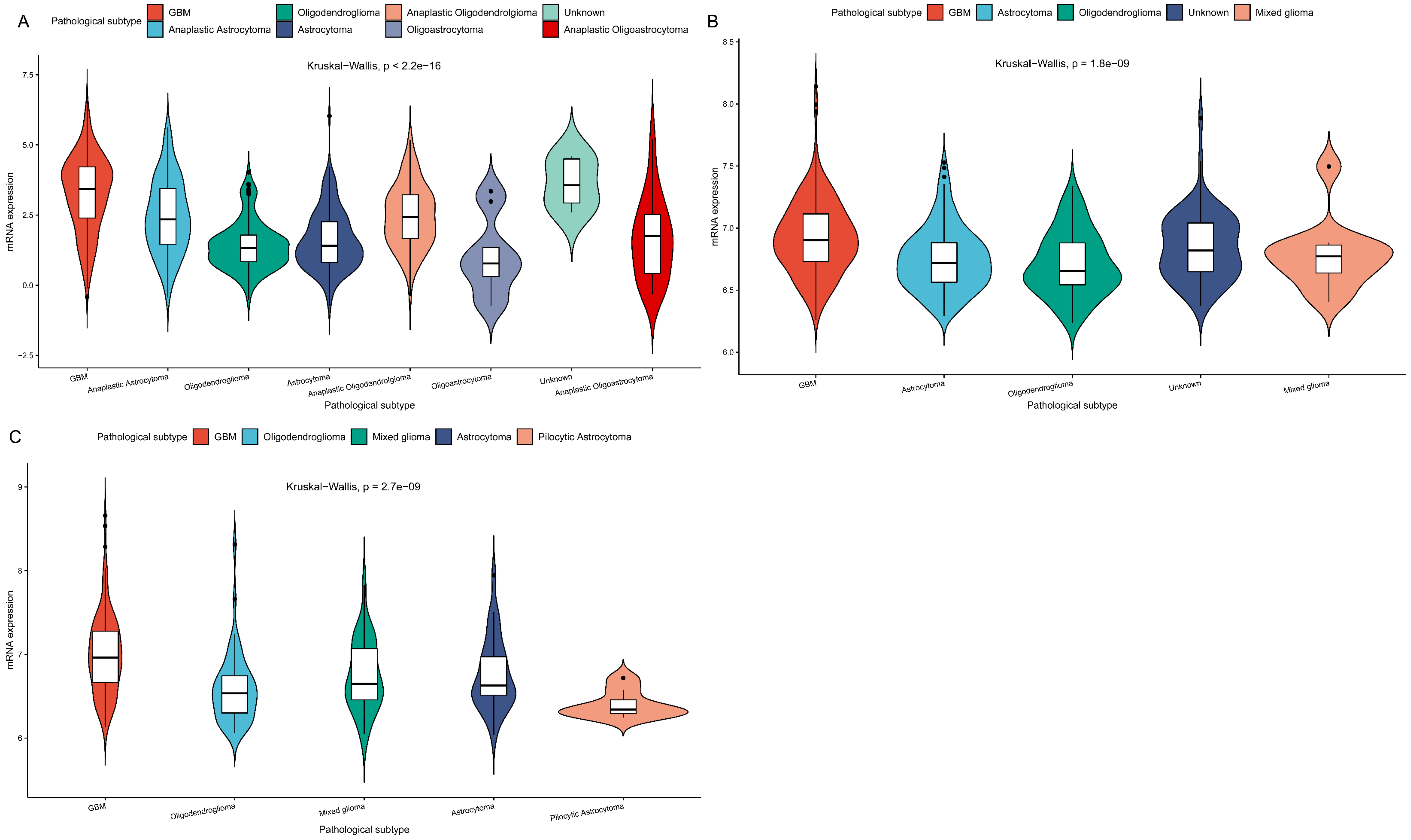

Figure S3. TK1 expression in various pathological subtypes. A. CGGA. B. Rembrandt. C. Gravendeel.

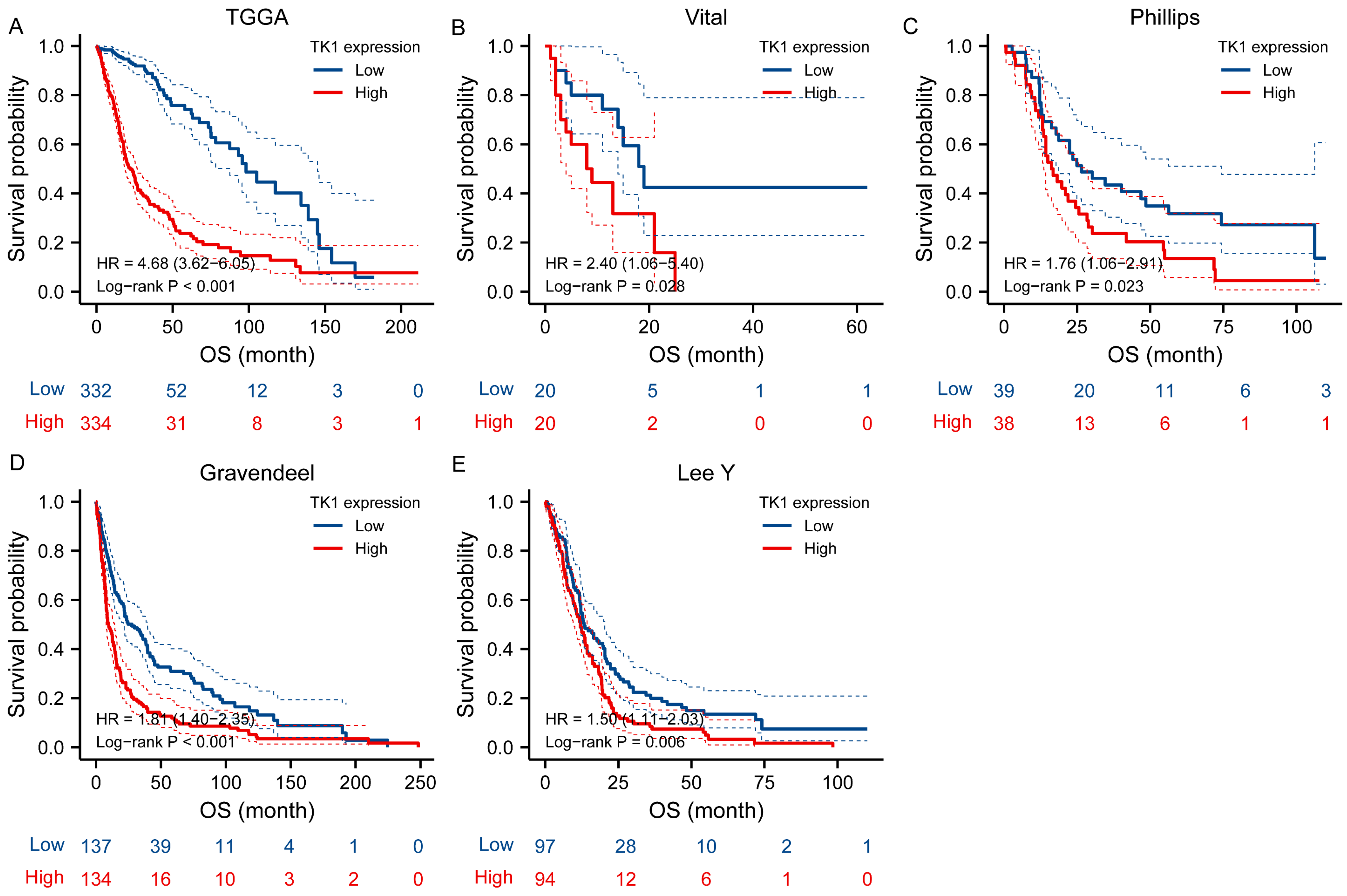

Figure S4. Kaplan–Meier analysis of overall survival of TK1 in five datasets. A. TCGA. B. Vital. C. Phillips. D. Gravendeel. E. Lee Y. Abbreviation: OS, overall survival.

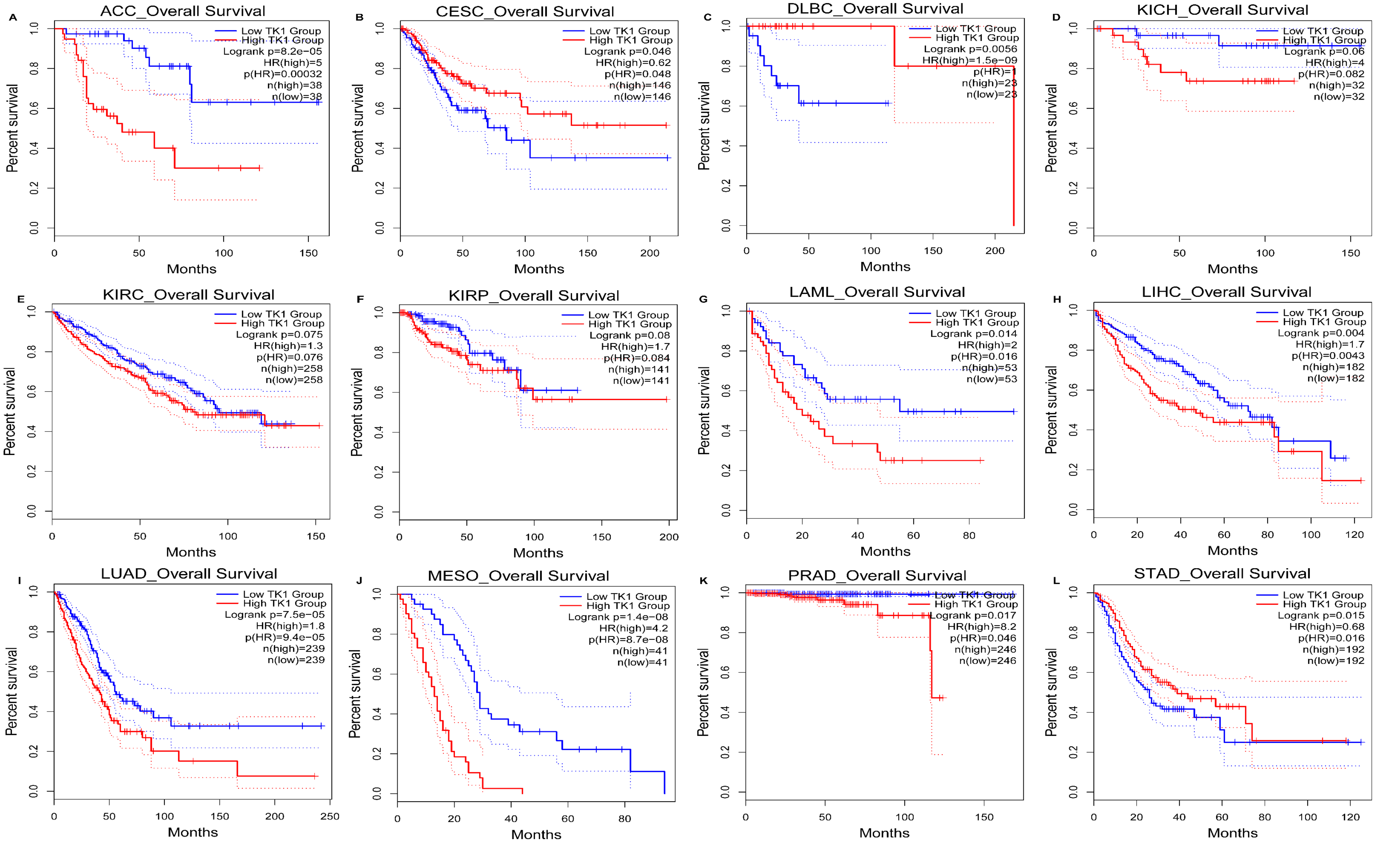

Figure S5. Kaplan–Meier analysis of overall survival of 12 cancer, including ACC (A), CESC (B), DLBC (C), KICH (D), KIRC (E), KIRP (F), LAML (G), LIHC (H), LUAD (I), MESO (J), PRAD (K), and STAD (L). Abbreviation: ACC, adrenocortical carcinoma; CESC, cervical squamous cell carcinoma and endocervical adenocarcinoma; DLBC, lymphoid neoplasm diffuse large B-cell lymphoma, KICH, kidney chromophob; KIRC, kidney renal clear cell carcinoma; KIRP, kidney renal papillary cell carcinoma; LAML, acute myeloid leukemia; LIHC, liver hepatocellular carcinoma; LUAD, lung adenocarcinoma; MESO, mesothelioma; PRAD, prostate adenocarcinoma; STAD, stomach adenocarcinoma.

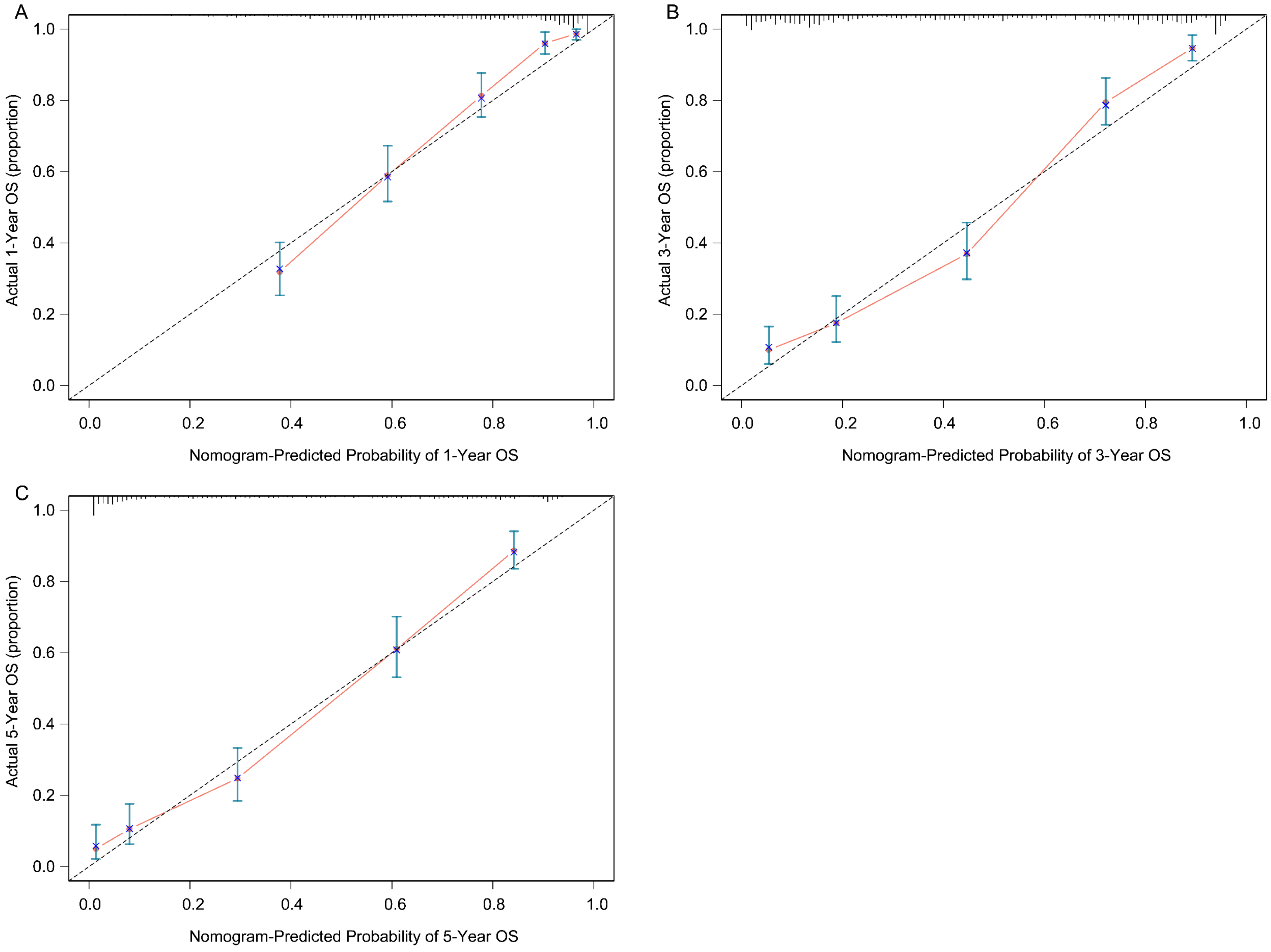

Figure S6. Calibration curve for nomogram to predict 1-year (A), 3-year (B), and 5-year (C).

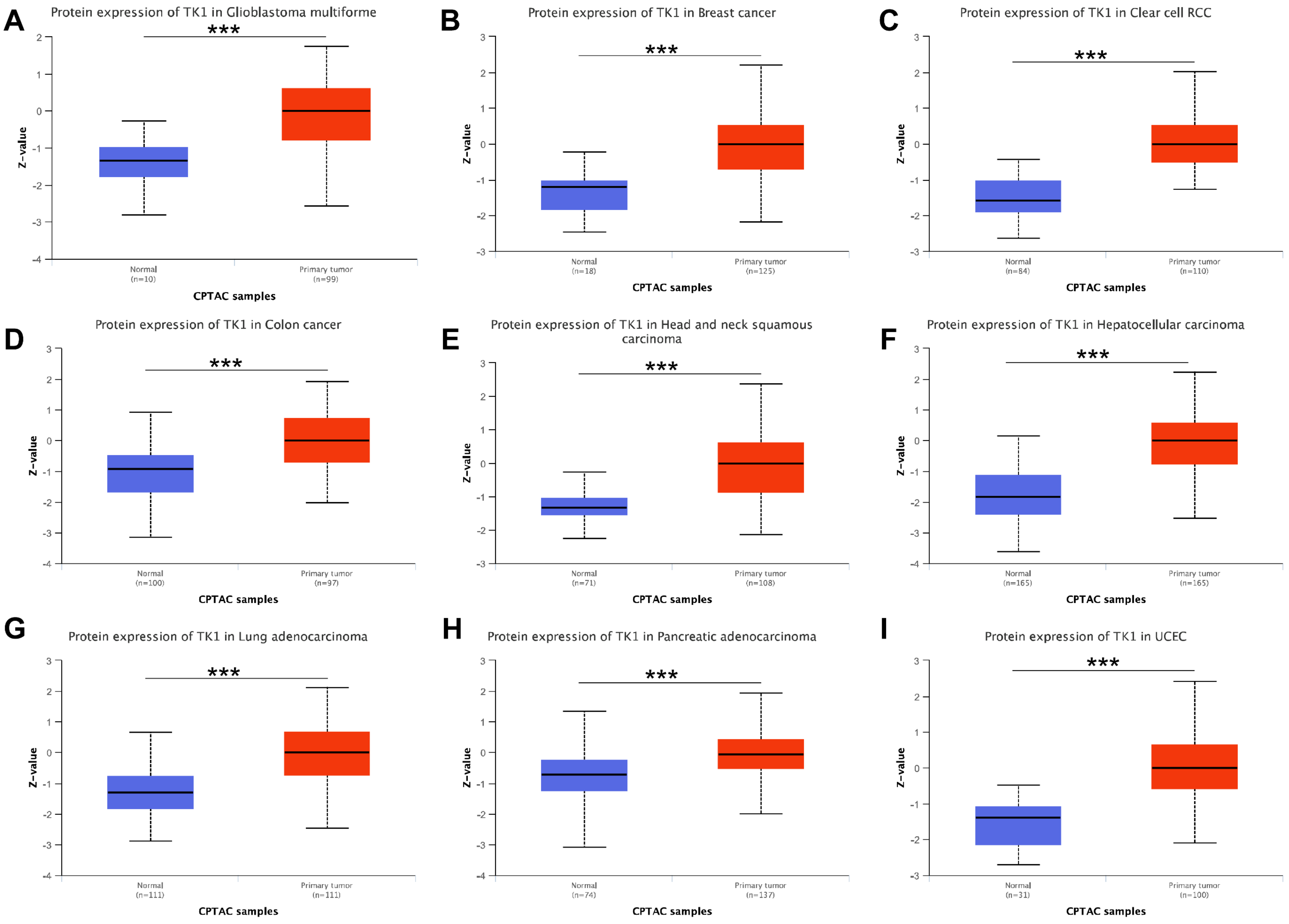

Figure S7. Upregulated protein expression of TK1 in glioblastoma (A), breast cancer (B), clear cell renal cell carcinoma (C), colon cancer (D), head and neck squamous carcinoma (E), hepatocellular carcinoma (F), lung adenocarcinoma (G), pancreatic adenocarcinoma (H), and UCEC (I) compared with normal tissue. $*p < 0.05$, $**p < 0.01$, and $***p < 0.001$. Abbreviation: RCC, renal cell carcinoma; UCEC, uterine corpus endometrial carcinoma.

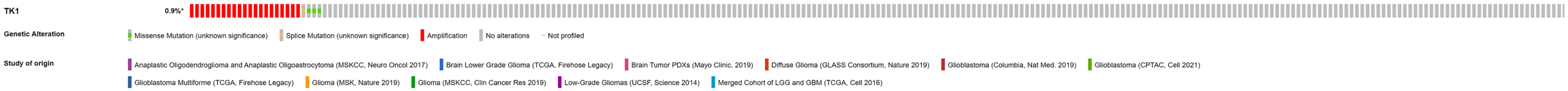

Figure S8. TK1 genetic alternations.

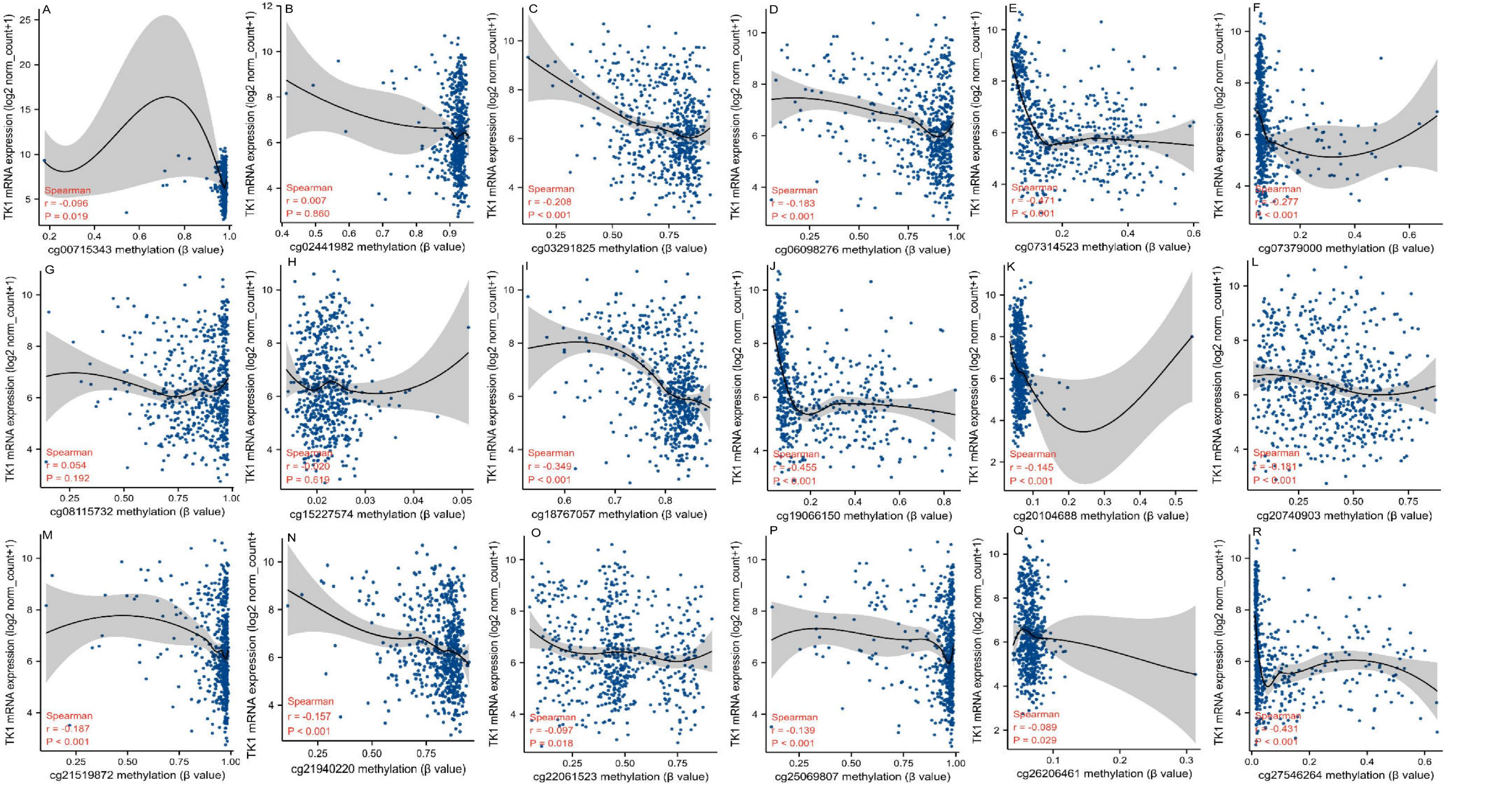

Figure S9. The relationship between TK1 mRNA expression and 18 TK1 DNA promoter CpG sites in glioma. A. cg00715343. B. cg02441982. C. cg03291825. D. cg06098276. E.cg07314523. F. cg07379000. G.cg08112732. H.cg15227574. I.cg18757057. J. cg1906650. K. cg20104688. L. cg20740903. M.cg21519872. N. cg21940220. O. cg22061523. P.cg25069807. Q. cg26206461. R. cg27546264.

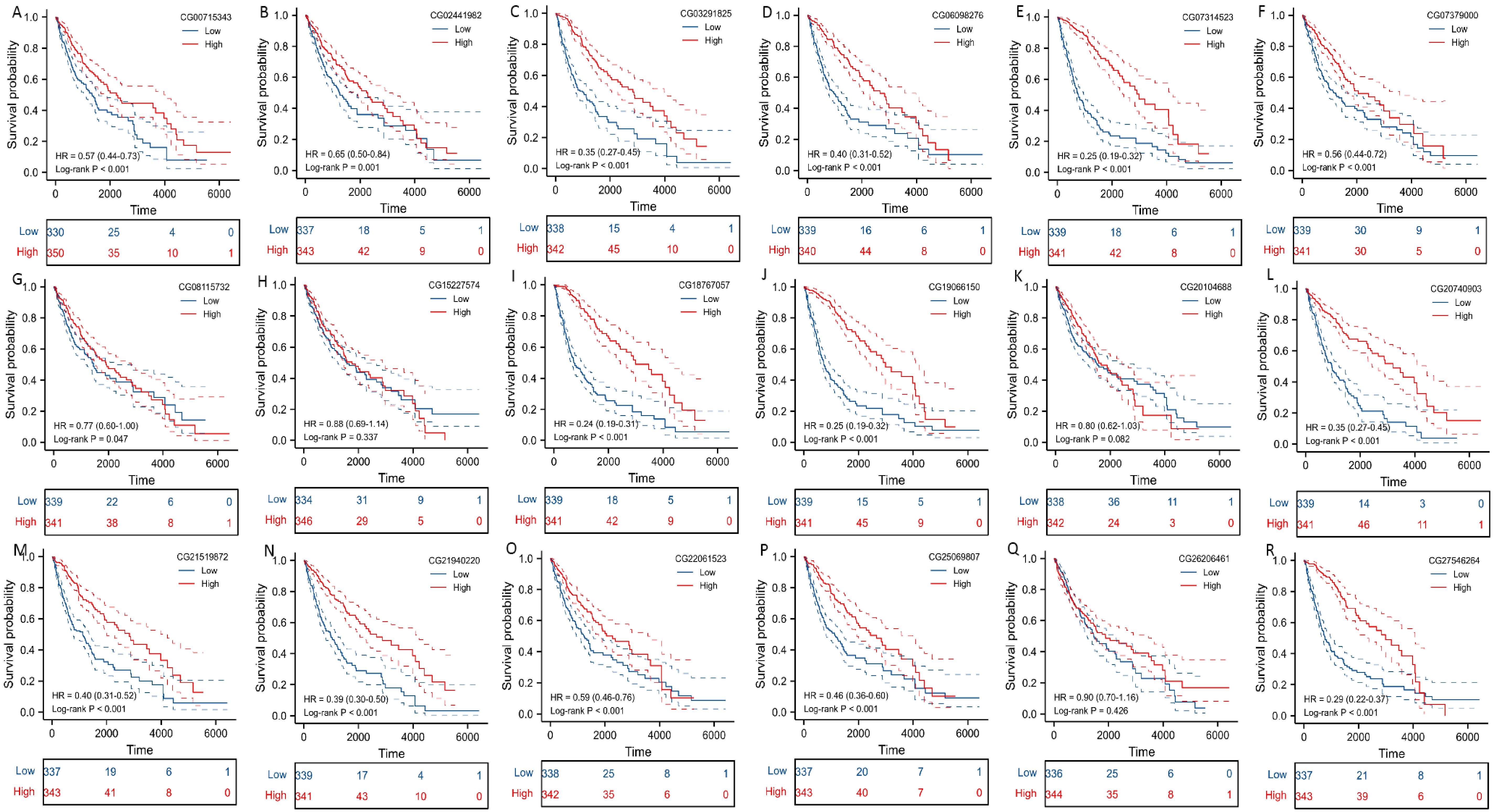

Figure S10. Kaplan-Meier curves of 18 TK1 DNA promoter CpG sites in glioma. A. cg00715343. B. cg02441982. C. cg03291825. D. cg06098276. E.cg07314523. F. cg07379000. G.cg08112732. H.cg15227574. I.cg18757057. J. cg1906650. K. cg20104688. L. cg20740903. M.cg21519872. N. cg21940220. O. cg22061523. P.cg25069807. Q. cg26206461. R. cg27546264.

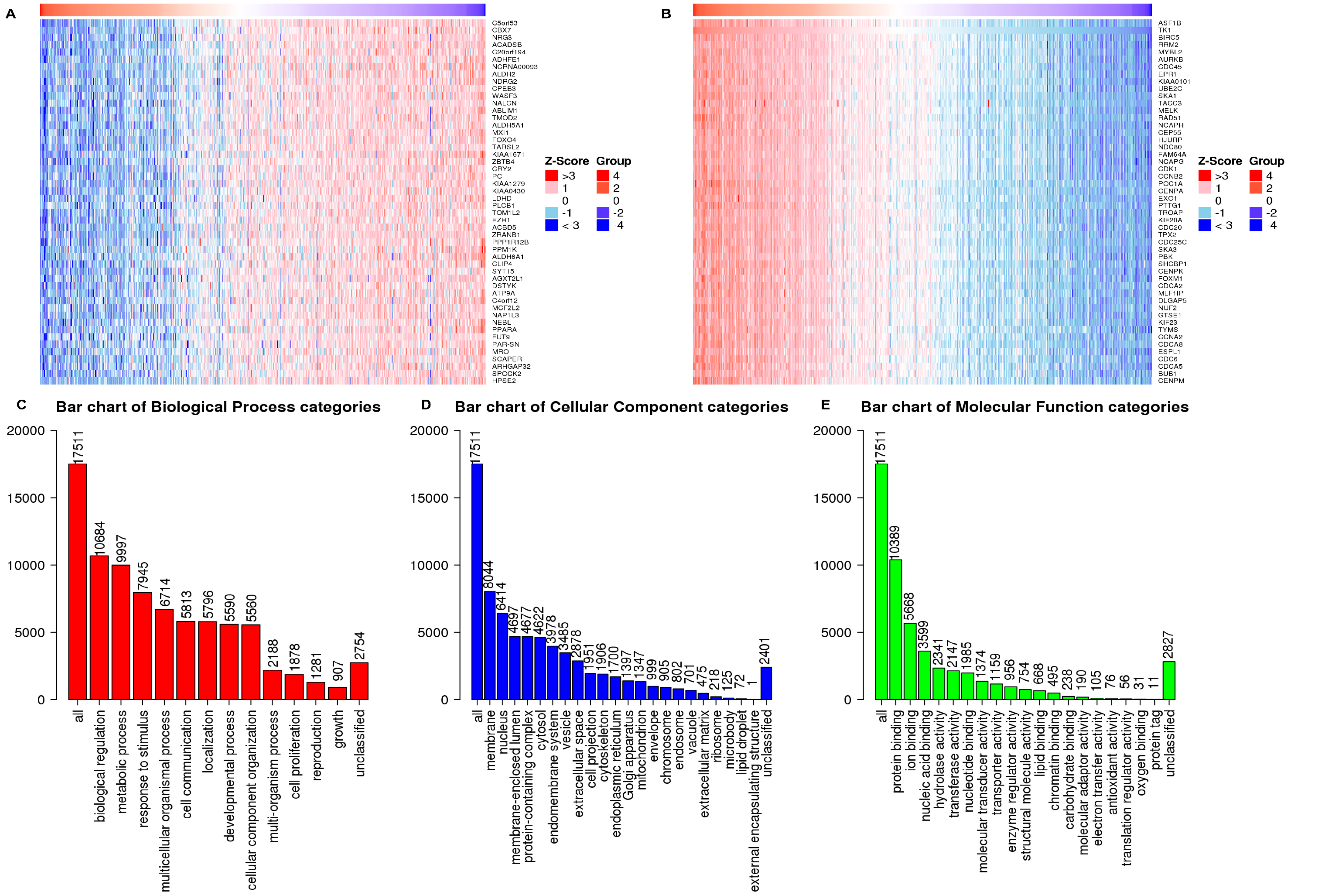

Figure S11. The co-expression genes of TK1 and GO functional annotation based on the TCGA_GBMLGG set. A. The heat map of top 50 negative associations with TK1. B. The heat map of top 50 positive associations with TK1. C-E. GO functional annotation involved biological process (C), cellular component (D), and molecular function (E).

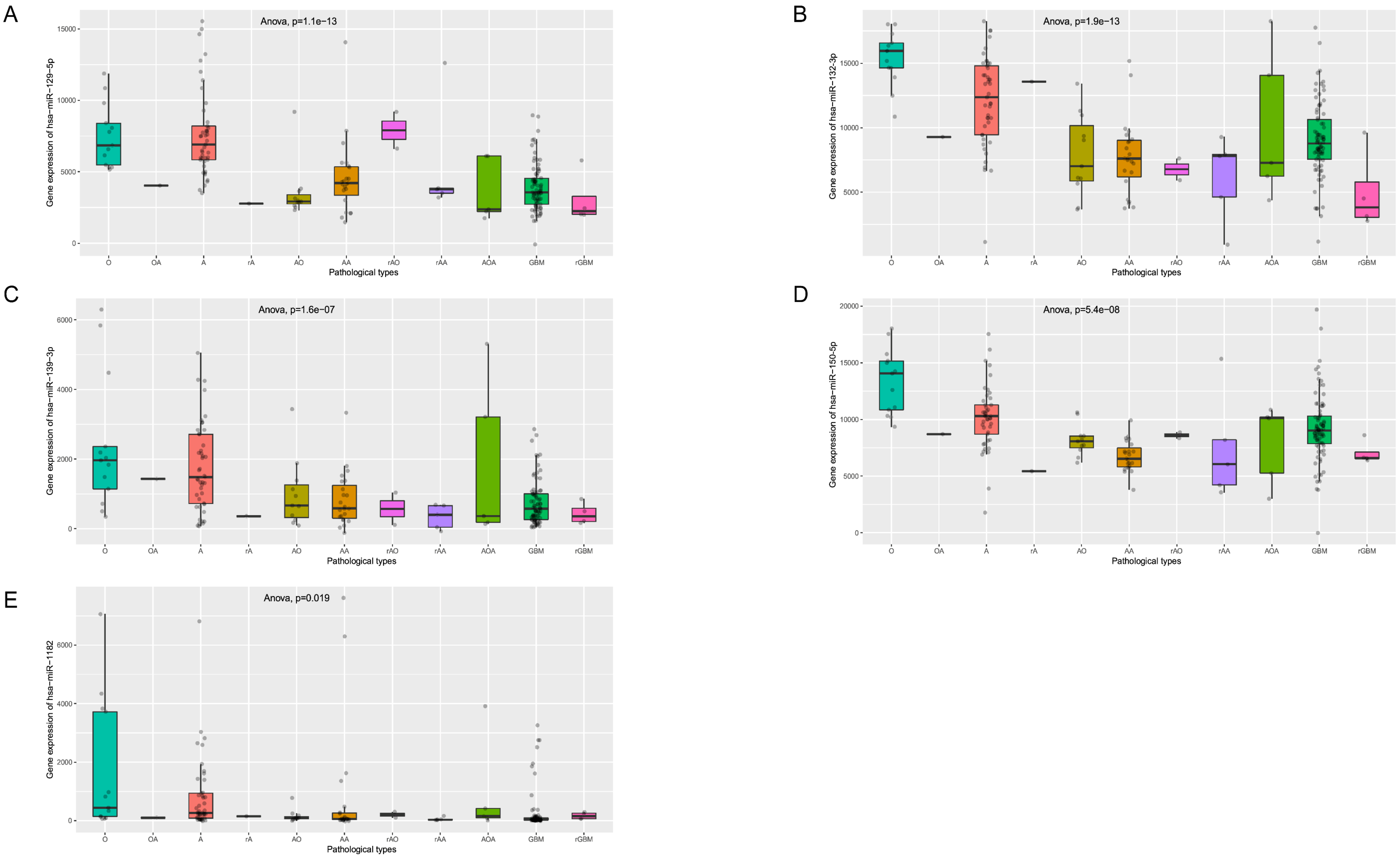

Figure S12. Five miRNA expression according to the pathological types of glioma. A. hsa-miR-129-5p. B. hsa-miR-132-3p. C. hsa-miR-139-3p. D. hsa-miR-150-5p. E. hsa-miR-1182. Abbreviation: GBM, glioblastoma; rGBM, recurrent glioblastoma; AA, anaplastic astrocytoma; rAA, recurrent anaplastic astrocytoma; AOA, anaplastic oligoastrocytoma; A, astrocytoma; rA, recurrent astrocytoma; O, oligodendroglioma; OA, oligoastrocytoma; AO, anaplastic oligodendrolgioma; rAO, recurrent anaplastic oligodendroglioma.
